# DNA Methylation and Transcription Patterns in Intestinal Epithelial Cells From Pediatric Patients With Inflammatory Bowel Diseases Differentiate Disease Subtypes and Associate With Outcome

**DOI:** 10.1053/j.gastro.2017.10.007

**Published:** 2018-02

**Authors:** Kate Joanne Howell, Judith Kraiczy, Komal M. Nayak, Marco Gasparetto, Alexander Ross, Claire Lee, Tim N. Mak, Bon-Kyoung Koo, Nitin Kumar, Trevor Lawley, Anupam Sinha, Philip Rosenstiel, Robert Heuschkel, Oliver Stegle, Matthias Zilbauer

**Affiliations:** 1 University Department of Paediatrics, University of Cambridge, UK; 2 Department of Paediatric Gastroenterology, University of Cambridge and Addenbrooke’s Hospital, Cambridge, UK; 3 European Molecular Biology Laboratory, European Bioinformatics Institute, Wellcome Genome Campus, Hinxton, Cambridge, UK; 4 Wellcome Trust - Medical Research Council Stem Cell Institute, University of Cambridge, Cambridge, UK; 5 Wellcome Trust Sanger Institute, Wellcome Trust Genome Campus, Hinxton, UK; 6 Institute of Clinical Molecular Biology (IKMB), Kiel University, Kiel, Germany

**Keywords:** Epigenetics, Intestinal Epithelium, Gut Microbiota, Human Intestinal Organoids, AC, ascending colon, AUC, area under the curve, CD, Crohn’s disease, DMP, differentially methylated position, DEG, differentially expressed gene, DMR, differentially methylated region, DNAm, DNA methylation, FDR, false discovery rate, IBD, inflammatory bowel disease, IEC, intestinal epithelial cell, MDS, multidimensional scaling, rDMR, regulatory DMR, ROC, receiver operator characteristic, SC, sigmoid colon, TI, terminal ileum, UC, ulcerative colitis, WGCNA, Weighted Gene Co-expression Network Analysis

## Abstract

**Background & Aims:**

We analyzed DNA methylation patterns and transcriptomes of primary intestinal epithelial cells (IEC) of children newly diagnosed with inflammatory bowel diseases (IBD) to learn more about pathogenesis.

**Methods:**

We obtained mucosal biopsies (N = 236) collected from terminal ileum and ascending and sigmoid colons of children (median age 13 years) newly diagnosed with IBD (43 with Crohn’s disease [CD], 23 with ulcerative colitis [UC]), and 30 children without IBD (controls). Patients were recruited and managed at a hospital in the United Kingdom from 2013 through 2016. We also obtained biopsies collected at later stages from a subset of patients. IECs were purified and analyzed for genome-wide DNA methylation patterns and gene expression profiles. Adjacent microbiota were isolated from biopsies and analyzed by 16S gene sequencing. We generated intestinal organoid cultures from a subset of samples and genome-wide DNA methylation analysis was performed.

**Results:**

We found gut segment-specific differences in DNA methylation and transcription profiles of IECs from children with IBD vs controls; some were independent of mucosal inflammation. Changes in gut microbiota between IBD and control groups were not as large and were difficult to assess because of large amounts of intra-individual variation. Only IECs from patients with CD had changes in DNA methylation and transcription patterns in terminal ileum epithelium, compared with controls. Colon epithelium from patients with CD and from patients with ulcerative colitis had distinct changes in DNA methylation and transcription patterns, compared with controls. In IECs from patients with IBD, changes in DNA methylation, compared with controls, were stable over time and were partially retained in ex-vivo organoid cultures. Statistical analyses of epithelial cell profiles allowed us to distinguish children with CD or UC from controls; profiles correlated with disease outcome parameters, such as the requirement for treatment with biologic agents.

**Conclusions:**

We identified specific changes in DNA methylation and transcriptome patterns in IECs from pediatric patients with IBD compared with controls. These data indicate that IECs undergo changes during IBD development and could be involved in pathogenesis. Further analyses of primary IECs from patients with IBD could improve our understanding of the large variations in disease progression and outcomes.

## Graphical abstract

**Figure:**
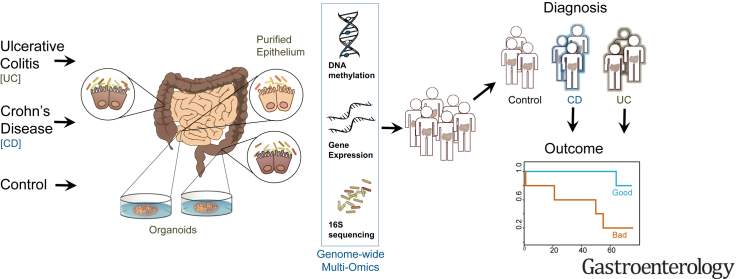

**Editor's Notes**

**Background and Context**

The intestinal epithelium is thought to play a critical role in the pathogenesis of inflammatory bowel Diseases (IBD), yet evidence derived from primary human tissue remain scarce.

**New Findings**

Purified intestinal epithelium from children newly diagnosed with IBD display distinct epigenetic and transcriptional alternations, which are partly retained in organoid cultures and correlate with disease outcome.

**Limitations**

Relatively small patient numbers require validation in additional cohorts.

**Impact**

Stable epigenetic alterations in the intestinal epithelium of children with IBD may explain variations in disease outcome and have potential to be developed into disease prognostic biomarkers in the future.

Inflammatory bowel diseases (IBD) cause chronic relapsing inflammation that can affect any segments of the digestive tract (ie, Crohn’s disease [CD]) or be restricted to the colon (ulcerative colitis [UC]).^1^, ^2^ Although these diseases can manifest at any age, approximately one quarter of patients^3^ are diagnosed in childhood or early adulthood, when the disease course and subsequent outcomes can be particularly severe.^3^

The etiology of IBD is multifactorial, although the interplay of factors is still poorly understood. Large-scale genome-wide association studies have helped to characterize the genetic risk, identifying over 200 disease-associated loci.^4^, ^5^ The striking overlap of genetic risk loci between CD, UC, and other immune-mediated diseases strongly suggests common immune regulatory pathways are affected in these conditions.^4^, ^5^ However, current estimates of the overall genetic contribution to IBD risk are still only 13% for CD and 8% for UC. The rapid increase in the incidence of IBD in recent decades,^6^, ^7^, ^8^ the stability of the human genome, the dysbiosis of the gut microbiome,^9^, ^10^, ^11^, ^12^ as well as epidemiologic evidence, all suggest an association between the rise in IBD and the recent changes in our environment.

Epigenetic mechanisms operate at the interface between genetic predisposition and our environment, capable of causing stable, potentially heritable changes of cellular function in response to environmental triggers.^13^, ^14^ Consequently, epigenetics is being increasingly recognized as a highly plausible mechanism that may both initiate and then maintain intestinal mucosal inflammation in human IBD. A growing number of studies have reported IBD-associated alterations in epigenetic profiles, as well as associated changes in gene expression and/or cellular function. For example, DNA methylation (DNAm) changes in mucosal biopsies and peripheral blood mononuclear cells of both adults and children diagnosed with IBD have been demonstrated.^15^, ^16^ However, the vast majority of studies were performed on mixed cell tissue samples (eg, whole blood, peripheral blood mononuclear cells, or mucosal biopsies) and, possibly because of changes in cellular composition, demonstrated a strong effect of inflammation on the observed epigenetic changes. Importantly, advances made by epigenetic consortia such as ROADMAP,^17^ BLUEPRINT,^18^ as well as single-cell RNA sequencing, have all demonstrated the importance of studying individual, disease-relevant cell types to best identify molecular alterations involved in pathophysiology.

Genetic and functional studies predominantly using mouse models and cell lines^19^, ^20^, ^21^ have provided strong evidence for impaired function of the intestinal epithelium in IBD. Yet, these models have done little to explain how the complex interplay between environmental factors, host genetics, intestinal cell function, and the adjacent microbiome lead to the development of the IBD phenotype and its subsequent evolution. To better elucidate specific alterations in this jigsaw, a genome-wide multi-layered omics approach of carefully selected primary cell samples is required. Importantly, in addition to unravelling novel aspects of disease pathogenesis, this approach in disease-relevant cell types (ie, the intestinal epithelium) could provide clinically relevant information. For example, in children and adults with IBD, it can be difficult to confidently distinguish CD from UC, with many patients remaining ‘unclassified’ despite disease progression. Intestinal epithelial cell (IEC)-specific ‘omics’ signatures have the potential to more rapidly and accurately diagnose the patient and, hence, improve the specificity of treatment management. Furthermore, variations of cell type-specific molecular profiles amongst IBD patients may be indicative of disease sub-phenotype and could therefore help to understand the large variations in disease behavior and outcome.

Therefore, we simultaneously profiled genotype, epi-genotype (ie, DNAm), gene expression, and the adjacent gut microbiota of highly purified IEC, obtained from children newly diagnosed with IBD and a matched cohort of non-IBD controls. We analyzed genome-wide ‘omics’ layers for potential IBD-specific alterations and functional consequences, as well as cross-talk between layers. Additionally, we generated intestinal epithelial organoids from patient biopsy samples and investigated their epigenetic profiles. Lastly, we applied statistical models to genome-wide datasets to test their ability to distinguish between disease subtypes, as well as potential correlation with disease outcome measures.

## Methods

### Patient Cohort

A cohort of 66 treatment-naïve children at diagnosis of their IBD, along with 30 age- and sex-matched non-inflammatory control children, were recruited by the Paediatric Gastroenterology team at Addenbrooke’s Hospital during 2013–2016. This study was conducted with informed patient and/or carer consent as appropriate, and with full ethical approval (REC-12/EE/0482). Sample and patient details are provided in Supplementary Tables 1 and 2. Children with macroscopically and histologically normal mucosa who had a diagnosis of IBD ruled out served as the non-disease control group. Each patient’s final clinical diagnosis was based on the revised Porto criteria.^22^ At the diagnostic colonoscopy, additional mucosal biopsies were taken from the small bowel (ie, terminal ileum [TI]) and 2 large bowel sections (ie, ascending colon [AC] and sigmoid colon [SC]). A blood sample was taken for patient genotyping. Clinical phenotype and outcome data were prospectively recorded over a minimum of 18 months post-diagnosis (Supplementary Table 1). The inflammation status of a sample (inflamed vs non-inflamed) was based on the histology of a paired sample taken within 2 cm of samples at the time of the initial endoscopy. Longitudinal samples were taken from the TI and SC of a subset of patients that underwent repeat endoscopy (CD: n = 14; UC: n = 9).

### Purification of Intestinal Epithelium

Biopsy samples were processed immediately and IECs purified using enzyme digestion and magnetic bead sorting for the epithelial cell adhesion molecule as described previously.^16^, ^23^ Mucus for the isolation of adjacent microbiota was collected during tissue processing from sieve and centrifugation supernatant, then pooled, pelleted, and stored at -80^°^C to extract DNA from the adjacent microbiota. Further information is provided in the Supplementary Methods.

### Human Intestinal Epithelial Organoid Culture

Intestinal organoids were generated from mucosal biopsies by isolation of intestinal crypts and culturing as described previously and detailed in the Supplementary Methods section and Supplementary Table 4.^24^

### DNA and RNA Extraction

DNA and RNA were extracted simultaneously from the same sample using the AllPrep DNA/RNA mini kit (Qiagen, Hilden, Germany). DNA from the adjacent microbiota was extracted using QIAamp DNA Stool Mini Kit and from whole blood using the DNeasy Blood and Tissue Kit (both Qiagen). DNA was bisulfite-converted using Zymo DNA methylation Gold kit (Zymo Research, Irvine, CA, USA).

### Arrays and Sequencing

Genome-wide DNA methylation was profiled using the Illumina Infinium HumanMethylation450 and EPIC BeadChip platforms (Illumina, Cambridge, UK; Accession Number: E-MTAB-5463). Sample numbers are provided in Supplementary Table S3. Expression profiling was performed using RNA-sequencing (RNA-seq) at the University of Kiel, Germany using an established pipeline as described previously.^10^ (Project accession number: E-MTAB-5464). Patient genotyping was performed using the Illumina OmniExpressExome-8 BeadChip Kit. 16S rRNA gene profiling of the adjacent microbiota was performed at the Wellcome Trust Sanger Centre (Hinxton, Cambridge). The 16S microbiota data can be found under EBI study ID PRJEB6663. For further details of the arrays and sequencing, please see the Supplementary Methods.

Locus-specific validation of DNA methylation profiles was performed on bisulfite-converted DNA after polymerase chain reaction amplification using the Pyromark Q24 (Qiagen) pyrosequencing system as described previously.^16^

### Bioinformatics Analyses

Extensive details of the bioinformatics methods used in this publication are described and referenced in the Supplementary Methods. Briefly, DNAm analyses were performed using minfi,^25^ sva,^26^ DMRcate,^27^ and limma^28^ R packages. RNAseq data was processed using established workflows.^10^ Microbiota composition analysis was performed using QIIME and phyloseq.^29^ Differential analysis was performed using limma^28^ for DNAm data (false discovery rate [FDR] <0.01) and DESeq2^30^ for gene expression data (FDR <0.01 and log fold change >±0.5). InnateDB^31^ and the Reactome pathways were used to perform pathway enrichment analysis for the disease signatures identified from omics data layers. The diagnostic potential of omics data layers was tested using random forest classification models. Diagnostic accuracy was assessed via area under the receiver operator characteristic curve (AUC), precision scores, and receiver operator characteristic (ROC) curves. Weighted Gene Co-expression Network Analysis (WGCNA)^32^ was applied to gene expression and DNAm data for each diagnosis (CD and UC) by gut segment to correlate omics datasets with clinical phenotypic variables.

## Results

### DNA Methylation and Gene Expression Profiling of Purified Intestinal Epithelium Reveals Gut Segment-specific and Disease-associated Alterations

**Figure 1:**
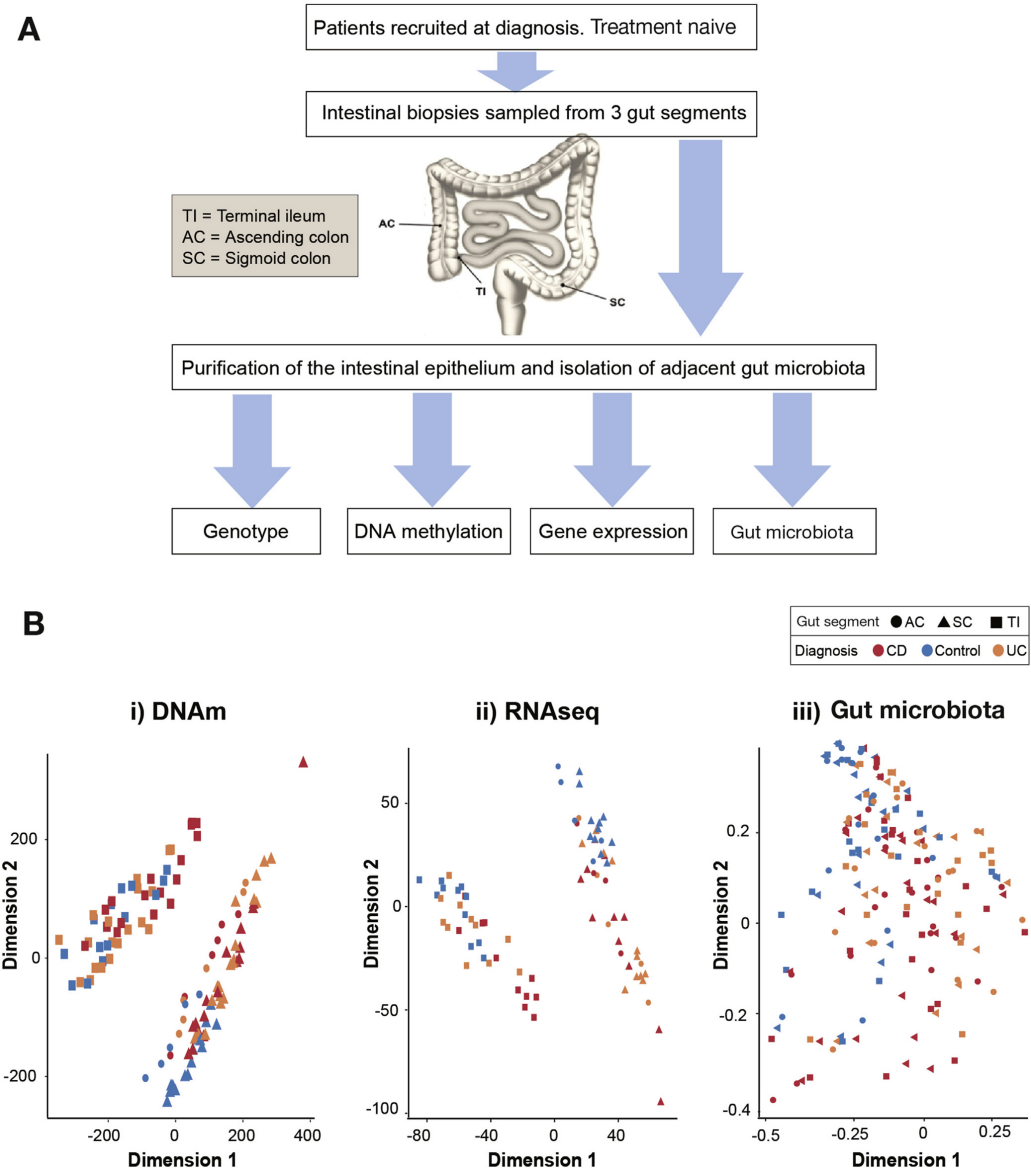
Overview of study design and multi-dimensional scaling (MDS) analysis of genome-wide datasets. (*A*) Outline of study design. (*B*) MDS plots for each dataset: (*i*) DNAm based on batch corrected M-values; (*ii*) r-log normalized RNAseq gene expression counts; (*iii*) gut microbiota 16S operational taxonomic units normalized counts. Samples are labelled according to diagnosis (CD, Crohn’s disease; UC, ulcerative colitis; control) and gut segment. Schematic in part *A* adapted from Tauschmann et al.^41^

To investigate IEC pathophysiology in pediatric IBD, we first performed unsupervised analysis of genome-wide DNAm, gene expression, and 16S microbial profiles generated from a total of 170 samples (Figure 1*A* and Table 1). Multidimensional scaling (MDS) plots indicate sample similarity/differences based on all data points included. MDS plots of DNAm and gene expression profiles revealed distinct clustering of samples by gut segment separating all TI-derived epithelium from colonic (ie, AC and SC) samples (Figure 1*Bi* and 1*Bii*). Moreover, samples derived from controls clustered closely both on an epigenetic (DNAm) and transcriptomic level for each gut segment. Interestingly, IBD-derived samples displayed more variation, with a subset of IBD samples distinctly separating from controls (Figure 1*Bi*, *Bii* and Supplementary Figure 1). In contrast to DNAm and gene transcription, no clear separation was evident from the 16S microbial community profiles using the same MDS approach (Figure 1*Biii*). However, analysis of the bacterial operational taxonomic unit by family abundance and alpha-diversity did reveal variation by gut segment (Supplementary Figure 2) and reduction in species diversity for CD patients (Supplementary Figure 2).

**Table 1:** Summary of Patients, Samples, and Generated Datasets

	DNAm			RNAseq			16S sequencing			Genotype
	TI	AC	SC	TI	AC	SC	TI	AC	SC	
Total samples at diagnosis	162			81			170			62
Total individuals	73	15	74	33	15	33	58	53	59	62
CD	31	5	32	11	5	11	21	21	22	24
450K cohort	13	5	13							
EPIC cohort	17		18							
Organoids	5		5							
UC	18	5	18	11	5	11	18	16	18	18
450K cohort	13	5	13							
EPIC cohort	5		5							
Controls	24	5	24	11	5	11	19	16	19	20
450K cohort	14	5	14							
EPIC cohort	3		3							
Organoids	7		7							
Repeat endoscopies										
UC	9		9							
CD	14		14							

AC, ascending colon; CD, Crohn’s disease; SC, sigmoid colon; TI, terminal ileum; UC ulcerative colitis.

Taken together, initial unsupervised analysis of genome-wide intestinal epithelial profiles reveals highly gut segment-specific signatures and suggests disease-associated alterations of DNAm and gene expression.

### Disease-specific Alterations in IEC Epigenetic and Transcriptional Profiles are Partly Independent of Inflammation Status

**Figure 2:**
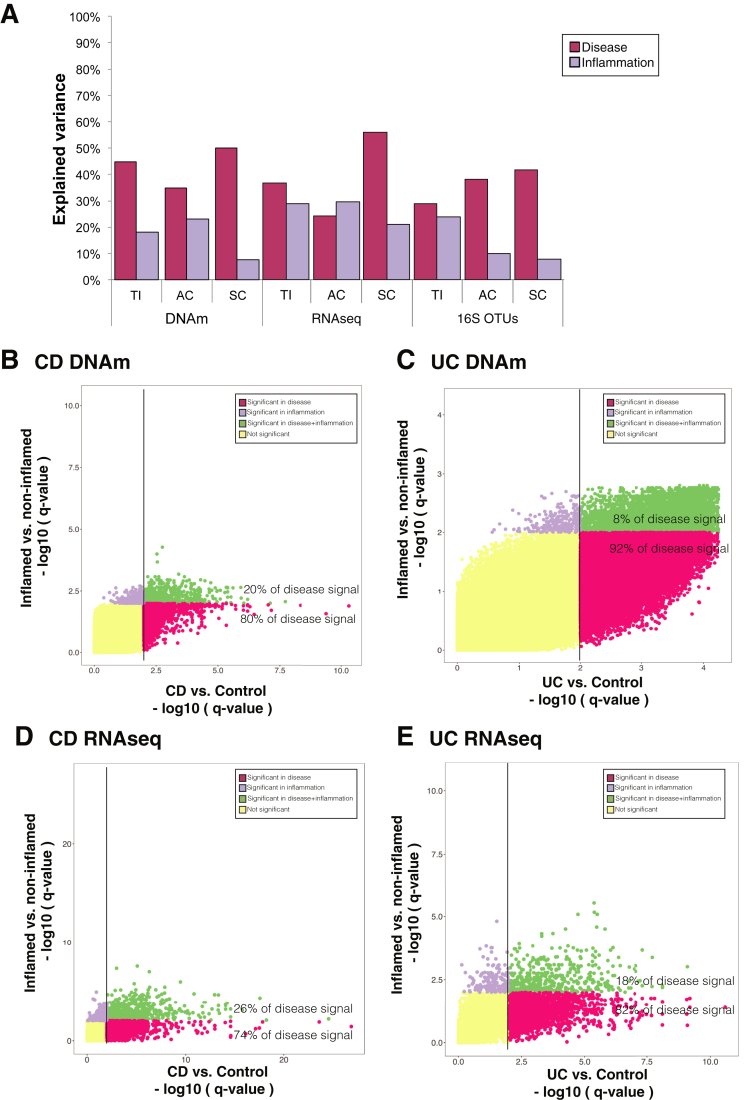
Contribution of diagnosis and inflammation to variance within each data layer. (*A*) Bar chart of the explained variance by diagnosis and inflammation across each dataset separated by gut segment. (*B*–*E*) Scatterplot of *P* values derived from differential DNAm (I and I) and gene expression (I and I) in sigmoid colon (SC) samples. For each CpG or gene, *P* values were generated for the comparison between Crohn’s disease (CD)/ulcerative colitis (UC) and control, and inflammation status (ie, inflamed vs non-inflamed). CpGs and genes with significant *P* values are plotted in purple for inflammation, in red for diagnosis, and in green if significant for both comparisons. Adjusted *P* < .01 was considered as significant.

Given the distinct clustering patterns we observed on a genome-wide scale, we next used a variance component model to assess the relative contribution of diagnosis and inflammation to the observed variance within each data layer, by gut segment. As shown in Figure 2*A*, the variation explained by disease (ie, diagnosis) exceeds that of inflammation in the majority of datasets. This highlights the presence of disease-specific molecular alterations, which are partly independent of the current inflammatory activity. Full results of the variance decomposition analysis can be found in Supplementary Figure 3. To extend these findings, we performed separate differential analysis (gene expression and DNAm) of inflammation and disease in the colonic epithelium. This allowed us to identify, for each CpG site or gene, the relative significance of inflammation and disease. Results showed that a majority of the differentially methylated positions (DMPs) between CD or UC and controls are primarily driven by diagnostic status and not inflammation (FDR <0.01, Figure 2*B* and *C*). Similarly, diagnosis explained 74% and 82% of the differentially expressed genes (DEGs) for CD and UC, respectively (Figure 2*D* and *E*).

Additionally, generating MDS plots by labelling samples according to gut segment, diagnosis, and inflammatory status did not show a clear separation between inflamed and non-inflamed samples (Supplementary Figure S1). Lastly, we also tested for the potential impact of inflammation on cellular composition in our purified samples by generating a gene expression heatmap of common epithelial and immune cell marker genes (Supplementary Figure S4). Although a number of genes were found to be differentially expressed between IBD and control samples (eg, DEFA5, DEFA6, LYZ, PLA2G2A, CD40, CD44), none of the marker genes correlated with inflammatory status, suggesting minimal impact of epithelial cell composition and immune cell contamination on the observed disease-specific molecular changes (Supplementary Figure S4).

In summary, these analyses demonstrate the presence of clear epigenetic, transcriptomic, and adjacent microbial alterations in the intestinal epithelium of children newly diagnosed with IBD, with a proportion being independent of intestinal inflammation.

### Differential Methylation Analysis Reveals Disease-specific Signatures That Affect Gene Transcription

Next we performed differential DNAm and gene expression analyses by comparing control, CD-, and UC-derived datasets for each gut segment. When performing these analyses, inflammation was controlled for within the differential analysis thereby allowing us to focus on molecular alterations that occur in relative independence of mucosal inflammation. Additionally, in an attempt to connect epigenetic and transcriptomic signatures, we identified differentially methylated regions (DMRs) that were located within 10kb of the transcription start site of a DEG. Such regions were termed regulatory DMRs (rDMRs).

**Figure 3:**
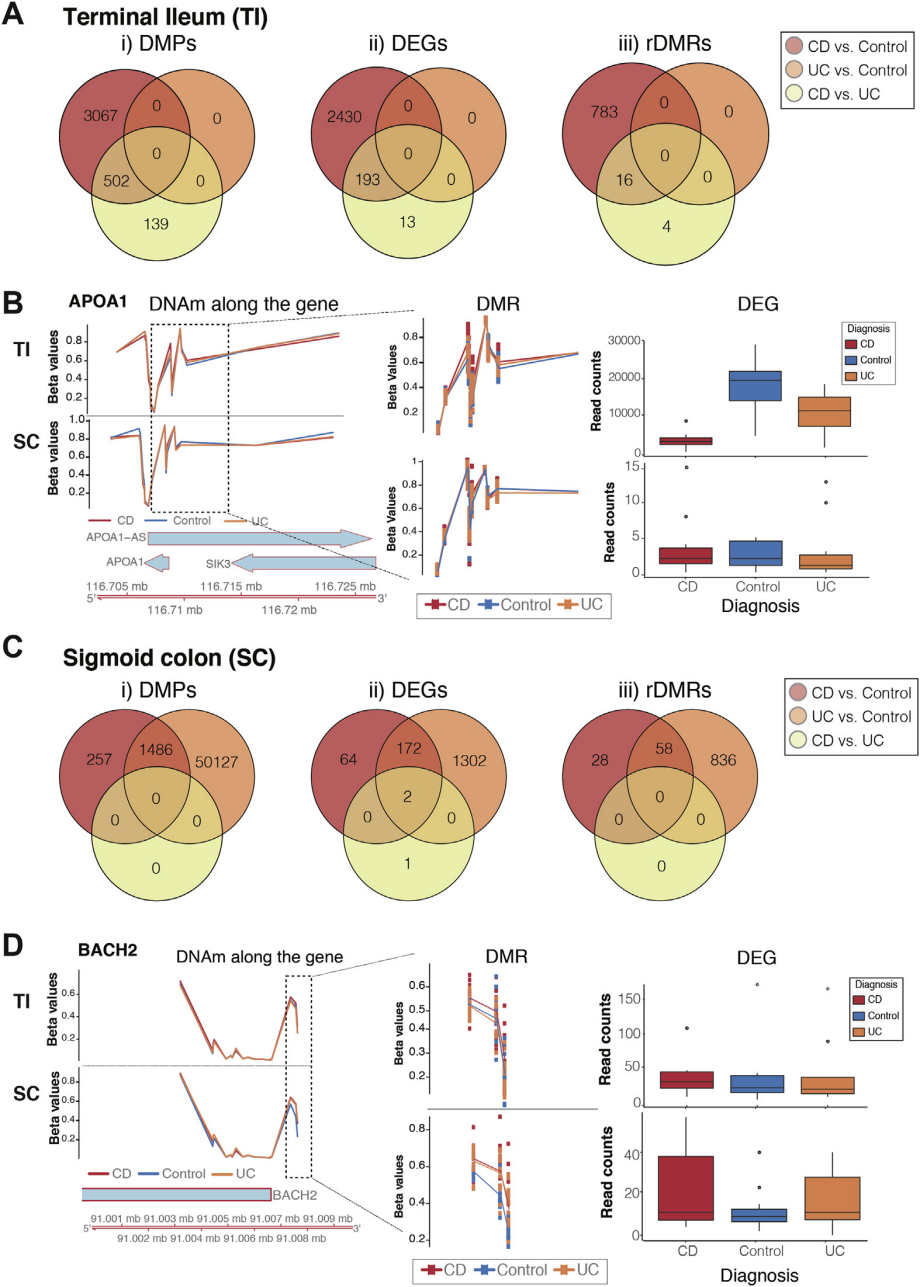
Differential DNAm and gene expression analysis were performed separately for terminal ileum (TI) (*A* and *B*) and sigmoid colon (SC) (*C* and *D*), taking mucosal inflammation into account. (*A* and *C*) Venn diagrams of significant differentially methylated positions (DMPs), differentially expressed genes (DEGs), and regulatory DMRs (rDMRs). (*B* and *D*) Example of disease-specific rDMRs displaying DNA methylation levels expressed as Beta value on the y-axis in the left panel separately for TI and SC samples in the upper and lower panel, respectively. Beta value of 0 represents un-methylated, while 1 represents fully methylated CpG site. Genomic location is indicated on the x-axis. The middle panel displays identified rDMR (enlarged). The right panel displays a boxplot of the respective gene expression according to diagnosis. (*B*) rDMR within the APOA1 identified in TI-derived epithelium of children diagnosed with CD. (*D*) rDMR within the BACH2 gene identified in colonic IEC.

Analysis of ileal IECs revealed CD-specific changes in both DNAm (Figure 3*Ai* and Supplementary Tables 5 and 6) and gene expression (Figure 3*Aii* and Supplementary Tables 7 and 8), when compared with either controls or UC, with a proportion overlapping between the 2 comparisons. In contrast, no significant DMPs or DEGs were identified when comparing UC with controls. Importantly, amongst identified rDMRs, several have previously been reported to be associated with IBD (eg, CASP1^33^ and APOA1^9^) (Figure 3*B*).

Contrary to the ileum, changes observed in the SC reflected a ‘common IBD’ signature, with a major overlap between UC and CD signatures and only a single significant DEG (RARRES3 [Retinoic Acid Receptor Responder 3]) identified between the 2 diagnoses (Figure 3*C* and Supplementary Tables 9–14). RARRES3 is thought to have growth inhibitory and cell differentiation activities. One example of an rDMR that jointly affects CD and UC in the colon is BACH2 (Figure 3*D*), a transcription regulator, where a decrease in DNAm matched the increase in gene expression levels in both CD and UC patients. Interestingly, a proportion of the CD-related changes identified in TI samples were also found to be present in SC samples (Supplementary Figure 5).

Overall, these results indicate that CD-specific DNAm and gene-expression changes are present in ileal IECs. In contrast, molecular changes observed in the colonic epithelium revealed a major overlap between CD and UC, reflecting a ‘common IBD’ signature.

### Pathway Enrichment Analysis of Identified rDMRs Reveal Both Common IBD and Disease-specific Pathways

**Figure 4:**
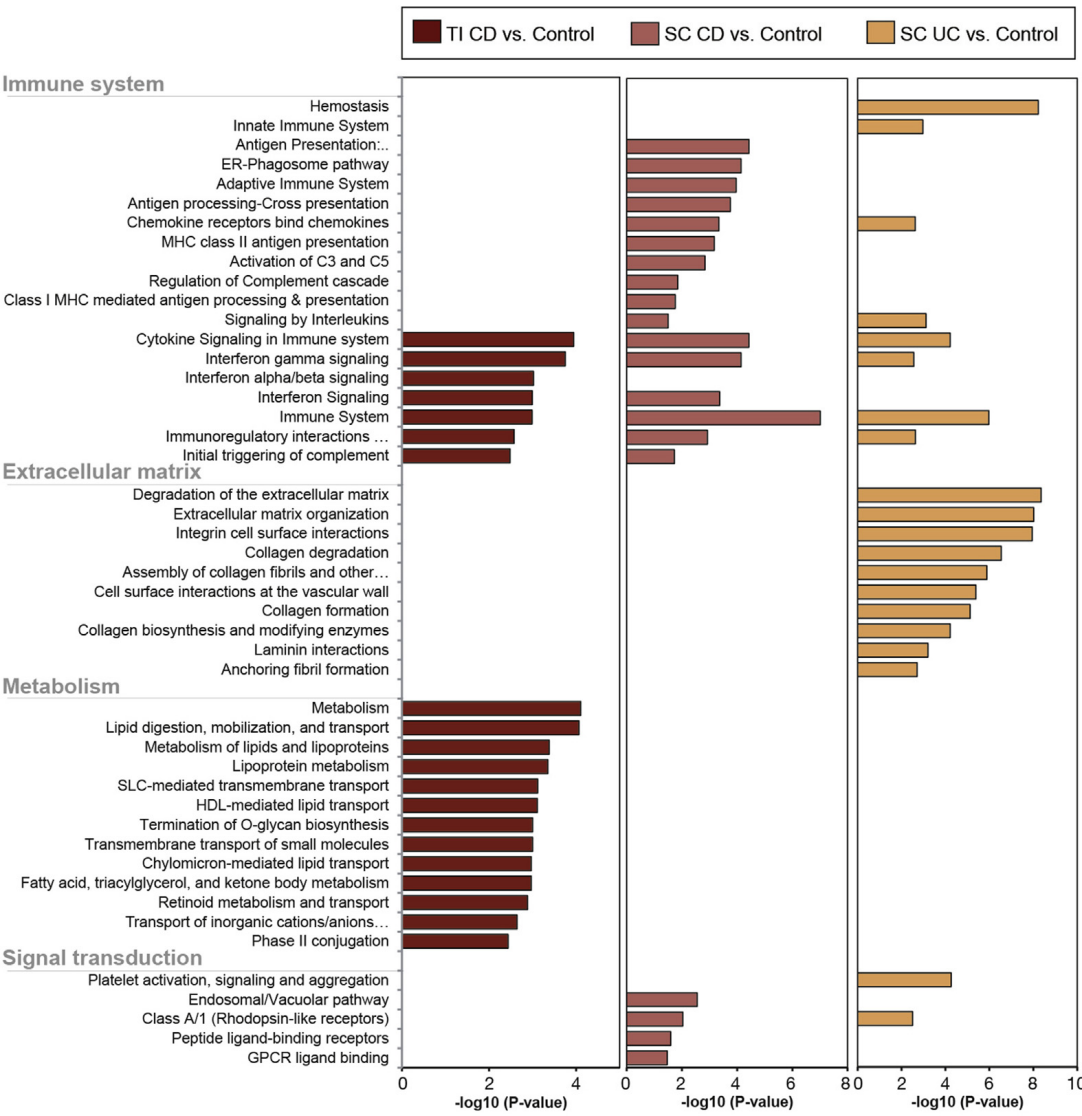
Pathway enrichment analysis of disease-specific regulatory DMRs (rDMRs). Pathway enrichment analysis was performed on identified rDMRs derived from the 3 comparisons between Crohn’s disease (CD) vs controls in terminal ileum (TI) and sigmoid colon (SC) samples (left and middle panel) and ulcerative colitis (UC) vs controls in SC samples (right panel). Analysis was performed using InnateDB and Reactome database and significant enrichment of individual pathways is displayed as the -log10 (adjusted *P* value).

The intestinal epithelium serves a wide range of functions as a physical, chemical and immunological barrier and a bridge between the innate and adaptive immune response.^34^ We used pathway enrichment analysis to investigate functional pathways (of rDMRs) that may be altered at diagnosis in a child with IBD. A wide variety of immune system-, metabolism-, and signal transduction-related pathways were significantly enriched (Figure 4). Many of the immune system-related pathways (eg, interferon signaling and immuno-regulatory interactions) are shared between the gut segments and diagnoses; suggesting common alterations are present in IBD. Moreover, several of the significantly enriched pathways have previously been implicated in either IBD pathogenesis or IEC function (Figure 4).

### IBD-associated intestinal Epithelial-specific Epigenetic Alterations are Stable Over Time and Partly Retained in Ex-vivo Organoid Culture

**Figure 5:**
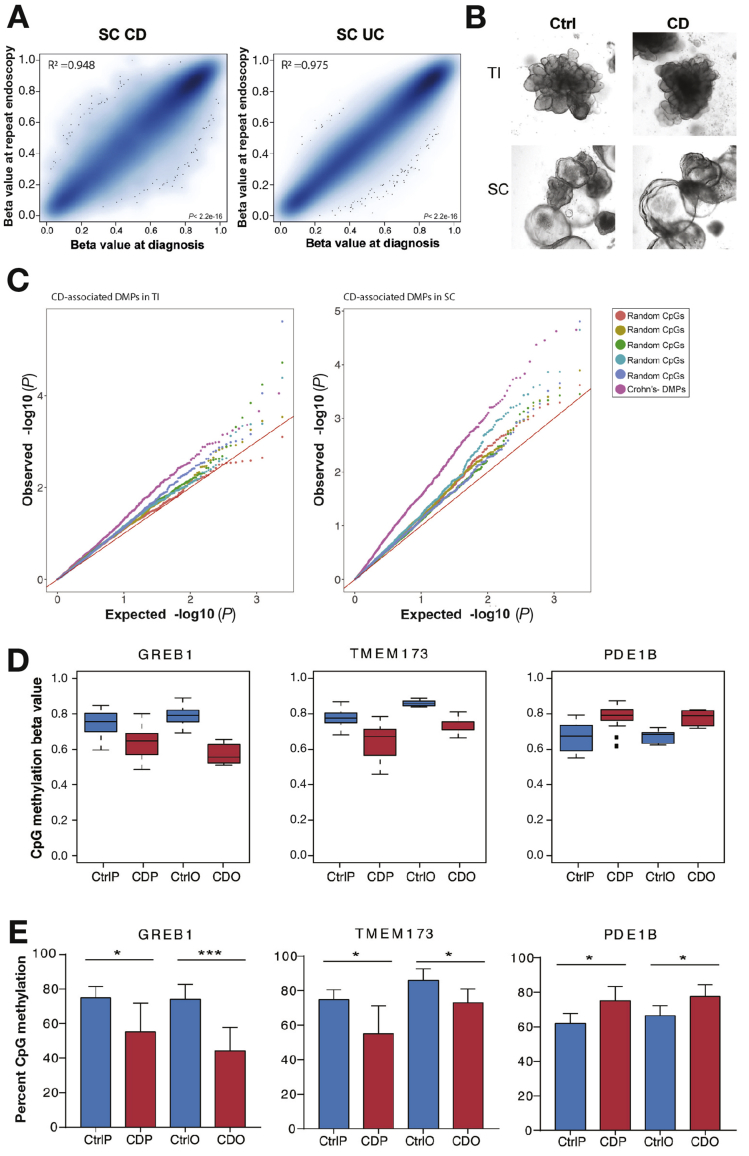
Stability of disease-associated intestinal epithelial DNA methylation changes: (*A*) Correlation plot of DNA methylation (Beta values) of disease-associated differentially methylated positions (DMPs) at diagnosis and at repeat endoscopy for each patient at the 2 time points. Shown are Crohn’s disease (CD)-associated DMPs (left) and ulcerative colitis (UC)-associated DMPs (right) in sigmoid colon (SC) epithelium (adjusted *P* <. 01). (*B*) Brightfield microscopic images of fully grown intestinal epithelial organoids derived from 2 gut segments (ie, terminal ileum [TI] and SC) of CD and control patients. (*C*) Quantile-quantile plot generated from organoid-derived genome-wide DNAm *P* values. Plotted are *P* values (observed vs expected) comparing specific CD-associated DMPs (from Figure 3) for each gut segment with randomly selected CpGs. (*D*) Examples of CD-associated DMPs being retained in patient-derived organoids. Plotted are beta values derived from genome-wide array data generated from purified colonic epithelium and respective organoids. GREB1, Growth Regulation By Estrogen In Breast Cancer 1; TMEM173, Transmembrane Protein 173; PDE1B, Phosphodiesterase 1B; CtrlP, Control purified IEC (n = 14); CDP, CD purified IEC (n = 13); CtrlO, Control organoids (n = 7); CDO, CD organoids (n = 5). (*E*) Validation of CpGs shown in *D*. Validation of genome-wide DNAm data using pyrosequencing. n = 5–7 per group; *P < .05; ***P < .001; unpaired, 2-tailed t-test between Ctrl and CD.

Next, we investigated the stability of IEC DNAm profiles in IBD patients over time. We obtained ileal and colonic biopsies (SC) from IBD patients both at diagnosis and at a later stage in their disease (n = 14 CD, n = 9 UC). Strikingly, CD- and UC-associated DMPs showed remarkable stability over time, demonstrated by the strong correlations of the methylation values at diagnosis and repeat endoscopy within each gut segment (Figure 5*A* and Supplementary Figure 6). This was in spite of changes to the underlying mucosal inflammatory status (see Supplementary Table 1). To further test the stability and potential inflammation independence of disease-specific epigenetic alterations in IBD-derived IEC, we generated patient-derived intestinal organoids from an additional cohort of children newly diagnosed with CD (n=5) and matched healthy controls (n=7). Expansion of mucosal crypts from TI and SC in culture gave rise to 3-dimensional organoids (Figure 5*B*). Organoids derived from CD patients did not differ in their microscopic appearance or culturing behavior from those derived from healthy controls (Figure 5*B*). However, assessing their genome-wide DNA methylation profiles revealed distinct alterations, suggesting that they retain a proportion of disease-associated epigenetic changes. Despite the relatively small sample number, CD-associated DMPs (ie, identified in Figure 3) showed a clear trend to be also differentially methylated in CD-derived compared with control organoids. This was indicated by the presence of inflated *P* values (larger difference between observed vs expected *P* values) of CD-associated DMPs compared with randomly selected CpGs (Figure 5*C*). Using locus-specific pyrosequencing, we were able to validate a subset of CD-specific DMPs that were retained in organoid cultures (Figure 5*D* and 5*E*).

Together, these data demonstrate that disease-associated epigenetic alterations in the intestinal epithelium are stable over time and are at least in part retained in ex-vivo organoid cultures.

### Intestinal Epithelial DMRs, DEGs, and rDMRs are Enriched Around Genetic IBD Risk Loci

Genome-wide association studies have successfully identified over 200 loci predisposing to IBD.^4^, ^5^ However, limited information is currently available on the potential mechanisms involved in mediating genetic risk and/or which cell types are particularly affected. Here we used our epithelial cell-derived molecular signatures to test for an enrichment of disease-specific DMRs, DEGs, and rDMRs within genomic IBD risk loci. We observed highly significant enrichment of DMRs, DEGs, and rDMRs in both colonic and ileal IECs for IBD risk loci, while limited enrichment was found for genetic variants that have been linked to other multifactorial diseases with an immune-mediated pathogenesis, such as Type 1 diabetes and multiple sclerosis (Supplementary Figure 7). Together these results suggest that interactions between the IBD risk loci and DNAm and/or transcription may occur in children carrying disease variants.

### IEC DNA Methylation and Gene Expression Signatures Accurately Predict Disease Status and Correlate With Clinical Outcome Measures

**Figure 6:**
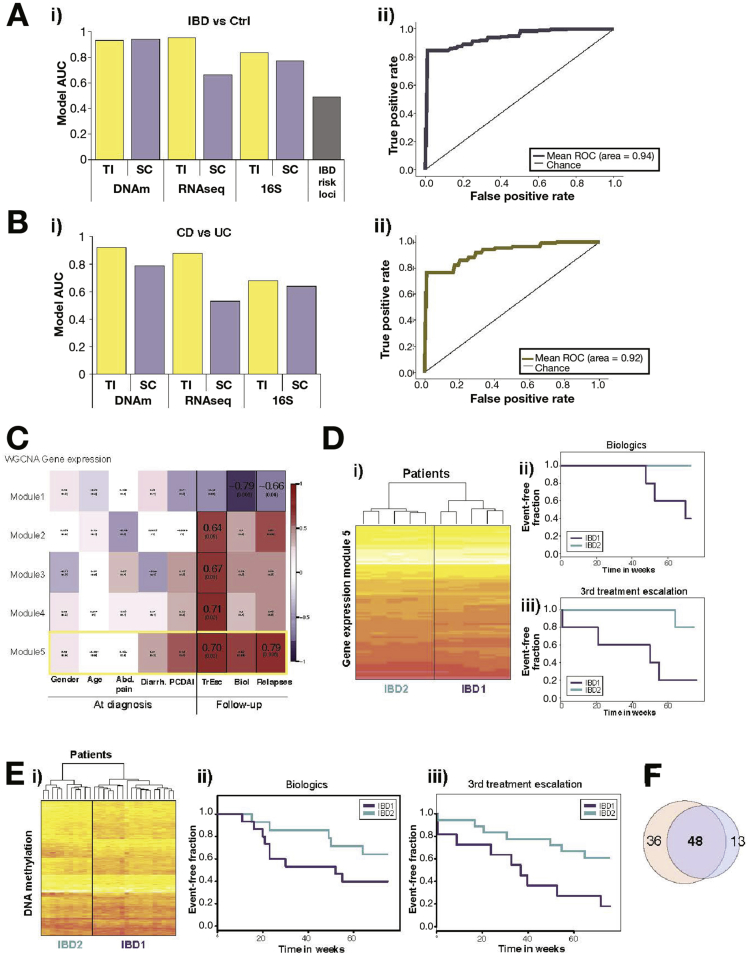
Correlation of intestinal epithelial cells (IEC)-specific molecular signatures with diagnosis and clinical outcome measures: IEC-derived epigenetic, transcriptomic, and microbial signatures were tested for their potential to predict diagnostic status (*A* and *B*) and correlation with disease outcome parameters (*C*–*F*). (*Ai*) Bar chart indicating area under the curve (AUC) of the best model to accurately differentiate samples based on diagnosis (ie, IBD vs controls). (*Aii*) ROC curve for the best diagnostic model (inflammatory bowel disease [IBD] vs control) using colonic DNAm data. (*Bi*) Bar chart of the AUC of the best models to differentiate between Crohn’s disease (CD) and ulcerative colitis (UC). (*Bii*) Receiver operator characteristic (ROC) curve for the best model separating CD from UC using ileal IEC DNAm data. (*C*) Weighted Gene Co-Expression Network Analysis (WGCNA) of CD terminal ileum (TI)-derived RNA-Seq data showing correlations between key gene-expression modules and clinical parameters. Each cell on the heatmap displays Pearson correlation coefficient and corresponding *P* value. Outlined cells indicate significant correlations. (*D*) Heatmap and hierarchical clustering of patients based on gene expression (ie, RNAseq counts) for strongest module. (*Dii* and *Diii*) Kaplan-Meier curves based on patient grouping derived from 7*Di*, ie, top gene expression module for use of biologics and time to third treatment escalation during 75 weeks of follow-up (n = 10 patients, *P* = .049 and *P* = .032, log-rank test). (*Ei*) Heat-map and hierarchical clustering of CpGs within strongest module identified by applying WCGNA to CD TI DNA methylation profiles. (*Eii* and *Eiii*) Kaplan Meier curves based on patient grouping derived from 7*Ei* for use of biologics (*Eii*) and time to third treatment escalation (*Eiii*) during 75 weeks follow-up (n = 29 patients, *P* = .025 and *P* = .043, log-rank test). (*F*) Venn-diagram showing the overlap between annotated genes that were present in the top modules for both gene expression and DNA methylation.

Given the striking IBD-associated changes observed in intestinal epithelial DNAm and gene expression, we went on to test the ability of these signatures to predict diagnosis. Additionally, we hypothesized that variation observed within IBD-derived patient samples could be indicative of future disease behavior and outcome. To address these hypotheses, we applied a machine-learning model (random forest) to the individual omics data layers. The model identified those data points (eg, CpGs, genes, operational taxonomic units) that could predict disease status for each patient with high precision and accuracy (see Supplementary Methods section for further details). As demonstrated in Figure 6, DNAm data derived from either gut segment produced a model with a high AUC (>0.8) (Figure 6*A*). The best model separating disease from control was based on DNAm data from the SC (AUC=0.94, cross-validation (CV)=40) with sensitivity of 75% and specificity of 100% (Figure 6*Ai* and 6*Aii*). Importantly, the use of ileal DNAm datasets allowed separation between CD and UC with high precision (77%), sensitivity (57%), and specificity (93%) (AUC=0.92, CV=24) (Figure 6*B*). The accuracy of the TI DNAm signatures in distinguishing CD and UC was confirmed in a follow-up patient cohort, analyzed using a second DNAm array platform (Illumina EPIC array, see Methods and Supplementary Figure 8). Full details of the models, including AUC, sensitivity, and specificity, can be found in Supplementary Table 15. In contrast, models built using the IBD risk loci from our patient genotyping data yielded the lowest model score (AUC=0.49) (Figure 6*Ai*).

To correlate genome-wide IEC profiles with clinical outcome measures (including binary, numerical, and categorical parameters) we used a Weighted Gene Co-expression Network Analysis (WGCNA) approach. WGCNA identifies patterns within a given dataset (ie, RNAseq data) and combines genes or CpGs that vary similarly across samples into modules. Each of these modules was then tested for a significant correlation with clinical outcomes. The application of WGCNA to RNAseq data derived from the TI of CD patients led to the identification of several gene modules (ie, groups of genes) that correlate significantly (correlation >±0.6, *P* < .05) with a number of disease outcome measures including the requirement for treatment with biologics and number of treatment escalations within the first 18 months following diagnosis (Figure 6*C*). Interestingly, modules correlating with disease outcome measures did not show any correlation with gender, age, or disease phenotype at diagnosis (eg, abdominal pain, diarrhea, and Pediatric Crohn’s Disease Activity Index). Clustering all samples according to expression levels of genes within the strongest modules separated CD patients in 2 groups (Figure 6*Di*). Kaplan Meier curves for these groups demonstrated striking differences in both the requirement for biologics and time to third treatment escalation (Figure 6*Dii* and 6*Diii*). In addition, we applied WGCNA to the DNAm data. Although the overall correlation of identified modules was less striking, separating samples according to DNAm profiles of the strongest module still demonstrated a significant difference in outcome measures between resulting patient groups (Figure 6*Ei*–6*Eiii*). Similar results were obtained from UC patient-derived signatures in SC samples (Supplementary Figure 9). Finally, comparing annotated genes from the top modules identified in RNAseq and DNAm datasets revealed an overlap of 57% and 79%, respectively, suggesting that expression signatures might be in part underpinned by stable epigenetic changes (Figure 6*F*). Based on these preliminary results, both DNAm and RNAseq data contain signatures that accurately predict disease status and correlate with selected disease outcome parameters.

## Discussion

Substantial evidence suggests that impaired function of the intestinal epithelium plays a major role in IBD pathogenesis. However, our current understanding of the exact mechanisms remains limited. It is also becoming increasingly clear that functional alterations in complex disease are likely to be caused by and/or result in a multifaceted interplay between several layers of cellular regulation. Specific to the GI tract, the intestinal microbiota adds further complexity because it has been shown to influence cellular function of the intestinal epithelium both in health and IBD.^9^, ^10^, ^12^ Given the wide range of phenotypes and diverse spectrum of disease behavior within the conditions we currently label as CD and UC, we urgently require novel molecular signatures to allow better classification of clinically relevant disease entities.

Here, we applied a multi-omics profiling approach to a highly purified IEC sample set obtained from a prospectively recruited, treatment-naïve, pediatric patient cohort. Unsupervised analysis revealed fundamental differences in the methylation and gene expression profiles by gut segment.^9^, ^10^, ^16^ Moreover, within each gut segment, we observed distinct disease-specific variation in both DNAm and gene expression, which were found to be partly independent of the presence or absence of microscopic mucosal inflammation. Specifically, we found that the majority of DNAm and RNAseq disease signatures from the SC were not primarily explained by inflammation status. This strongly suggests that there are underlying epigenetic and transcriptomic changes within IECs in IBD patients, which are present irrespective of inflammatory activity. Our findings further expand on previous studies using whole gut biopsies, which reported major transcriptional or epigenetic changes that were primarily associated with the presence of mucosal inflammation.^9^, ^10^ Although we also observed a strong, inflammation-associated signal, purification of the intestinal epithelium (and thereby removal of infiltrating immune cells) has allowed identification of a cell type-specific signature that does not seem to be exclusively driven by mucosal inflammation.

In contrast to genome-wide DNAm and gene transcription profiles, unsupervised MDS analysis of our 16S data did not show any specific sample clusters and/or clear association with key phenotypes such as gut segment, disease entity or inflammation. This is most likely because of the large inter-individual and intra-individual variation; an observation that has been previously reported by others.^9^, ^11^, ^12^ However, supervised analyses revealed gut segment as well as disease-associated changes in microbial composition. Analysis of the 16S data in combination with the epithelial omics data was unable to identify strong correlations between DEGs and 16S abundances or dose-dependent relationships for subgroups of patients. Nevertheless, we consider the fact that our 16S data was generated from microbes isolated from individual gut segments as novel and therefore potentially highly valuable as reference for future work in this rapidly evolving field.

Further investigating disease-specific DNAm and gene expression changes, we were able to identify a number of significant DEGs and DMRs, a proportion of which overlapped (rDMRs), indicating a functional interconnection between the 2 data layers. Reassuringly, a number of identified genes had previously been reported, including APOA1^12^ and CASP1.^9^ When comparing identified DMRs, DEGs, and rDMRs, we discovered that significant changes in the TI were only present in CD-derived samples. In contrast, analysis in the colonic epithelium showed both CD- and UC-specific changes, which also displayed a major overlap likely reflecting common phenotypic features shared between the 2 conditions. Together these data suggest the presence of a CD-specific signature in the TI epithelium and a common IBD signature in the SC. The identification of shared, enriched pathways for the 2 diagnoses further supports this hypothesis. Additionally, enrichment for pathways implicated in the cross-talk between cells of the innate and adaptive immune response highlights the important role of the intestinal epithelium in orchestrating intestinal host defense and suggests that alterations in these key processes may lead to the initiation and/or persistence of gut inflammation in IBD.

The impact of the observed IEC-specific epigenetic alterations on IBD pathogenesis will depend at least in part on the stability of such molecular signatures. Investigating IEC DNAm profiles of the same patient at 2 time points (ie, at diagnosis and at later disease stage) allowed us to demonstrate the strikingly high stability of disease-associated methylation signatures in small bowel and colonic IEC. This was despite changes in medication and mucosal inflammation over a period of up to 20 months. These findings suggest that stable epigenetic alterations may contribute to chronic relapsing inflammation by mediating altered IEC function. Interestingly, CD-derived epithelium appeared to retain a degree of disease-specific alterations even when cultured ex-vivo as organoids, further highlighting both their stability and relative independence of mucosal inflammation. Additionally, our findings add further support to recent reports on patient-derived intestinal organoids to be used as novel translational research tools.^35^

Despite the major success of genome-wide association studies in identifying disease-predisposing genetic loci, information on the functional consequences and cell specificity remain limited. Expression quantitative trait loci have been identified for a subset of the IBD risk loci from whole biopsies^36^ and blood cell subsets.^37^ More recently, differences in DNAm and chromatin conformation^38^ were also identified for a subset of the IBD risk loci in immune cells.^39^ Our study adds further detail and specificity by demonstrating enrichment of disease-specific DMRs, DEGs, and rDMRS within IBD susceptibility loci^4^, ^5^ in both gut segments.

An additional major strength of our prospectively recruited pediatric patient cohort was the availability of detailed phenotype and disease outcome data, allowing us to test for potential correlation between molecular signatures and clinical phenotypes. Despite the relatively small sample numbers included in these analyses, both the potential diagnostic and prognostic value of our IEC signatures is evident. While current diagnostic approaches are sufficient for most patients, a minority of patients requires repeated and prolonged investigations to confirm a diagnosis. Additionally, it is frequently challenging to differentiate UC from CD in children, both at diagnosis and later in the disease course. Therefore, a diagnostic model to reliably differentiate CD from UC, such as the model built using DNAm data from the ileum with high sensitivity and specificity, could be of clinical value. Correlating genome-wide molecular signatures with clinical outcome measures continues to be a major challenge and a wide range of bioinformatics tools have been developed. We decided to utilize WGCNA, which has been successfully applied to both RNA-seq and DNAm datasets, allowing identification and correlation of individual gene modules with clinical parameters.^40^ Results are highly encouraging because we discovered a number of gene expression modules that correlated strongly with the number of relapses and the requirement for treatment with biologics. Interestingly, overlapping modules derived from applying WGCNA to RNAseq data with those derived from DNAm data revealed a major overlap, suggesting prognostic expression signatures maybe at least in part underpinned by epigenetic changes.

As a limitation to our study, we acknowledge that the total number of patients included is relatively low and hence some of the analyses performed, particularly those that correlate signatures with clinical outcome, should be considered as preliminary. However, to the best of our knowledge this is the first and largest study applying a multi-omics profiling approach to a unique sample collection of highly purified intestinal epithelium. The fact that all patients were recruited at diagnosis (treatment-naïve) also represents an important strength. Last but not least, although our study was performed on a pediatric patient cohort, we consider our findings to be equally relevant to adult-onset IBD given the similarities in disease phenotype (particularly in teenage onset) and common concepts of disease pathogenesis.

In summary, our study is the first to apply a multi-omics profiling approach to a large collection of purified intestinal epithelial samples. The findings clearly demonstrate disease-specific abnormalities in epithelial cell function in children with IBD. We also highlight how specific data signatures might be indicative of disease status and behavior and therefore have a potential to be of clinical relevance in the future.
