# Supplementary material for: DNA Methylation and Transcription Patterns in Intestinal Epithelial Cells From Pediatric Patients With Inflammatory Bowel Diseases Differentiate Disease Subtypes and Associate With Outcome
Source: Gastroenterology. 2018 Feb;154(3):585–98. doi: 10.1053/j.gastro.2017.10.007 (PMC6381389; doi:10.1053/j.gastro.2017.10.007)
Supplement: Supplementary Methods [file mmc1.pdf]

Supplementary Methods

Patient cohort

A cohort of 66 treatment-naïve children at diagnosis of their IBD (CD=43, UC=23), along with 30 age- and sex-matched non-inflammatory control children, were recruited by the Paediatric Gastroenterology team at Addenbrooke's Hospital during 2013-16. This study was conducted with informed patient and/or carer consent and with ethical approval (REC-12/EE/0482). Children with macroscopically and histologically normal mucosa served as the non-disease control group (n=30). Each patient's final clinical diagnosis was based on the revised Porto criteria[1–3]. At diagnostic colonoscopy, mucosal biopsies were taken from the small bowel (i.e. Terminal Ileum = TI) and two large bowel sections (i.e. Ascending Colon=AC and Sigmoid Colon = SC). The inflammation status of a sample (inflamed vs. non-inflamed) was based on the histology of a paired sample taken within 2 cm of samples at the time of the initial endoscopy. Longitudinal samples were taken from the terminal ileum and sigmoid colon of a subset of patients that underwent repeat endoscopy (CD n=14, UC n=9).

A blood sample was taken for patient genotyping. Clinical phenotype and outcome data was prospectively recorded over a minimum of 18 months post-diagnosis. Supplementary Table S1 provides clinical details and sample availability for each patient.

These samples were used for several omics analyses including DNA methylation arrays (Illumina HumanMethylation450 and Illumina EPIC beadchips), RNAseq, 16S sequencing and genotyping (Illumina OmniExpressExome-8 beadchip), the methods of which are described below. Table 1 outlines the samples analysed by each method.

Purification of intestinal epithelium

Biopsy samples were processed immediately and IECs purified using enzyme digestion and magnetic bead sorting for the epithelial cell adhesion molecule (EpCAM) as described previously[4,5]. Briefly, samples were washed with Hank's balanced salt solution and incubated with an enzyme mix of liberase (Roche) and hyaluronidase (Merck) at 37°C on a horizontal shaker. The resulting cell suspension was passed through a 40 µm sieve, spun down, resuspended and stained with anti- Epithelial Cell Adhesion Molecule (EpCAM) magnetic microbeads with added Fc-receptor block (both Miltenyi Biotec). After washing and resuspension, a magnetic automated cell separation system (autoMACS, Miltenyi Biotec) was used to positively select for EpCAM(+) cells. Sorted EpCAM(+) cells were resuspended in RLT Plus lysis buffer (Qiagen) supplemented with 1% β-Mercaptoethanol (Sigma), then passed through a homogenizing column (Qiagen) and stored at -80°C until further processing. Mucus for the isolation of adjacent microbiota was collected during tissue processing from the sieve

and centrifugation supernatants, then pooled, pelleted and stored at -80°C to extract DNA from the adjacent microbiota. Quality of epithelial separation was routinely assessed by flow cytometry confirming high purity as described previously[4,5].

DNA and RNA extraction

DNA and RNA were extracted simultaneously from the same sample using the AllPrep DNA/RNA mini kit (Qiagen). DNA from the adjacent microbiota was extracted using QIAamp DNA Stool Mini Kit and from whole blood using the DNeasy Blood and Tissue Kit (both Qiagen). DNA was bisulfite- converted using Zymo DNA methylation Gold kit (Zymo Research).

Arrays and sequencing

Patient genotyping was performed using the Illumina OmniExpressExome-8 BeadChip Kit.

Genome-wide DNA methylation was profiled using two available array platforms; the Illumina Infinium HumanMethylation450 BeadChip or Illumina EPIC platform (Illumina, Cambridge, UK). An initial cohort was processed on the Illumina Infinium HumanMethylation450 BeadChip including purified epithelium from three gut segment samples and a subset of longitudinal samples. Additional samples were processed on the Illumina EPIC BeadChip including additional purified epithelial samples from the ileum and colon, longitudinal samples and organoid samples. Accession Numbers: E-MTAB-5463. An overview of sample numbers can be found in Table 1 and supplementary Table S3.

Expression profiling was performed using RNA-sequencing (RNA-seq). RNA integrity was checked using an Agilent Bioanalyzer and mRNA was sequenced at the University of Kiel, Germany using an established pipeline as described previously[6]. Project accession number: E-MTAB-5464.

16S rRNA gene profiling of the adjacent microbiota was performed at the Wellcome Trust Sanger Centre (Hinxton, Cambridge). Samples were PCR-amplified using barcoded fusion primers targeting the V1-V2 region of the gene (27f_Miseq and 338R_MiSeq) and sequenced on the Illumina MiSeq platform using 2×250 bp cycles. The 16S microbiota data can be found under EBI study ID PRJEB6663.

Supplementary Table S2 lists patients and array identifiers for all data layers.

DNA methylation analysis

Both K450 and EPIC methylation arrays provide a quantitative measure for DNA methylation at single CpG sites (>450,000 and >850,000 sites respectively) across the genome. DNA methylation (DNAm) analyses were performed using *minfi*[71], *sva*[8], *DMRcate*[9] and *limma*[10] R packages. First, functional normalisation[5] was applied using a strict detection threshold ($P <$ 1E-5). For each CpG site, beta values were calculated from the ratio of methylated (M) and unmethylated (U) probes from the array, B=M/(M+U+100), which range from 0 to 1 (0=non methylated, 1=fully methylated). Additionally, M-values were calculated as log(M/U). M-values were used for statistical analysis as they are normally distributed, beta values were used for visualisation of the data only. We applied ComBat, (*sva* package[8]) to the beta and M-values at the individual chip level, also including covariates (gender and inflammation) from the phenotypic data when stated. DNAm probes with known SNP co-localization or presence on the sex chromosomes were excluded using *DMRcate* (rmSNPandCH: settings maf=0.05, distance=2, rmXY=True)[9]. This protocol was the same for both DNAm datasets (i.e. samples profiled on the K450 and EPIC platform). A total of 423,394 CpG sites were retained in 450K array and 748,362 In EPIC array. Combining K450 and EPIC datasets was performed before normalisation and batch correction. A total of 374,213 CpGs were retained which are present on both arrays. DMRs were identified from the M-values using *DMRcate* (default parameters) and plotted using beta values and the *Gviz*[11] R package. DNAm results were annotated using the *IlluminaHumanMethylation450kmanifest*[12] R package for 450K array data and *IlluminaHumanMethylationEPICanno.ilm10b2.hg19* R package for EPIC array data. Figure 1-4, 5D, 6A-B and the DMPs used for Figure 5A-C are based on analysis of purified IEC at diagnosis using 450K array (n=41 patients). Figure 5A and 6C-F include further samples both at diagnosis and repeat endoscopy (measured on 450K or EPIC platform). All organoids were profiled using EPIC arrays.

RNAseq analysis

RNAseq data was processed by filtering out low quality residues (fastq_illumina_filter - ,http://cancan.cshl.edu/labmembers/gordon/fastq_illumina_filter/) and adapters were trimmed (cutadapt[13]); the filtered reads were mapped to the human genome (Grch37) with tophat2 (parameters " --library-type fr-firststrand --b2-very-sensitive") (tophat2[14], bowtie[15] and samtools[16]). The GTF file (GRCh37, release 19 GENCODE) for the alignment was downloaded from GENCODE. The raw read-counts per gene were generated using htseq-count[17]. R-log normalised counts for gene expression levels were calculated using *DESeq2*[18].

Microbiota composition

The 16S rDNA profiling and analysis was performed using the QIIME protocol[19–23] and *phyloseq*[24] R package. Operational taxonomic units (OTUs) were picked using the *Greengenes* database (updated November 2015). OTUs identified in the negative control samples were removed from all samples as likely contaminants.

Differential analysis and variance analysis

Only autosomes were considered for variance analysis and the identification of differential features. Multidimensional scaling analysis (MDS) was applied to examine sample relationships in different assays (normalised data DNAm: batch corrected M-values; RNAseq: r-log normalised read counts; 16S: normalised OTU counts). These analyses were based on an Euclidean sample distance matrix calculated from the individual omics datasets (cmdscale, dist R function), which was then used to generate coordinates per sample for low dimensional representation of the samples. This was then combined with phenotypic data to label the samples based on key phenotypic information. Differential analysis was performed using *limma* for the DNAm data on both the batch-corrected M-values and the batch+covariate corrected M-values, thereby assessing differences between the disease groups tested e.g. IBD vs. Control. All tests were carried out for individual CpG sites, following the Benjamini- Hochberg stepdown procedure to adjust for multiple testing (significant effects at adjusted $P < .01$ were reported). *DESeq2*[18] was used to test for differential gene expression using read counts, comparing disease group (using ± age and inflammation as covariates). As before Benjamini-Hochberg was used to adjust for multiple testing and reported significant differentially expressed genes at adjusted $P< .01$ for a minimum log fold change of ±0.5. Alpha and Beta diversity calculations were performed on the 16S OTU counts using phyloseq[24].

Variance decomposition was performed using a linear mixed model, considering each dataset (normalised data DNAm: batch corrected M-values; RNAseq: r-log normalised read counts; 16S: normalised OTU counts) and each gut segment (autosomes only) separately. For each data point (e.g. CpG sites, genes), we fit random effects to estimate the variance attributable to age, gender, disease and inflammation as four key phenotypic variables in these datasets. Parameters were estimated using (restricted) maximum likelihood, thereby estimating the proportion of variance explained by each of these factors (Figure 2A). The lead phenotype was chosen as the phenotype contributing the greatest proportion of the variance (Figure S3). The average explained variance for each phenotype was calculated based on the lead phenotype for each data point.

Pathway enrichment analysis

InnateDB[25] was used to perform pathway over-representation analysis for the disease signatures identified from our omics layers including rDMRs, using the Reactome pathways as the null set. For each data layer the annotated gene name and adjusted $P < .01$ from the differential analysis were used. Pathway analysis was performed using a hyper-geometric test and the Benjamini- Hochberg correction.

Human intestinal epithelial organoid culture

Intestinal organoids were generated from mucosal biopsies by isolation and culturing of Intestinal crypts in a protocol adjusted from Sato et al[26]. Freshly obtained TI and SC biopsies were washed with PBS and incubated in 2.5.mM EDTA at 4°C for 30 min. Crypts were released by pipetting with a P1000 pipette, spun down, seeded in Matrigel extracellular matrix (Corning) and covered in culture medium as detailed in Table 4. Conditioned media were kindly provided by the tissue culture facility at the Wellcome Trust-MRC Stem Cell Institute, University of Cambridge, UK. The Wnt-producing L-cell line was kindly provided by Hans Clevers (Hubrecht Institute, NL). The Rspo I producing cell line was kindly provided by Calvin Kuo (Stanford University, CA, USA). Organoids were maintained by medium change every 2 days and by splitting every 7-10 days via mechanical disruption.

Human intestinal epithelial organoid analysis

Differential analysis was analogous to that of data from the primary cohort; see above. This analysis was restricted to the set of N=374,213 probes present on both array formats, to enable cross-array platform comparisons. P-values from differential analysis of the organoids (n=12) were used to compare the DMPs from the purified epithelium disease comparisons (e.g. SC: CD vs. Control and TI: CD vs. Control) to five random selections of CpG sites from across the genome. QQ plots were used to show the observed vs. expected p-values from the random CpG selections and the disease associated DMPs derived from purified, primary epithelium. Locus-specific validation of DNA methylation profiles was performed on bisulfite-converted DNA after PCR amplification using Pyromark Q24 (Qiagen) pyrosequencing system as described previously[5].

Genetic Enrichment

Significant disease signatures from each gut segment and omics layer were compared to IBD risk loci and additional disorders (Alzheimers; Multiple Sclerosis (MS) and Type 1 Diabetes Mellitus (T1D)). A set of n=225 IBD risk loci were obtained from[27–29] while disease loci for other diseases were taken from DisGeNET (v4.0 (http://www.disgenet.org/, Nov 2016) all SNP-

disease associations list[30,31]. SNPs were chosen based on the above disease selection, presence in GWASCAT and publication after 2005.

We assessed the number of disease variants that were in proximity to at least one rDMR or DEG (distance window 10kb and 1mb for rDMRs and DEGs respectively). To assess the chance expectation of this overlap we used SNPsnap[32] to generate n=1000 sets of random non-risk loci elsewhere in the genome, where each variant was matched for allele frequency (±5%), gene density (±50%), distance to nearest gene (±50%) and a distance cut-off of r^2=0.5 for linkage disequilibrium (default SNPsnap settings) and excluding the HLA locus. The number of SNPs tested for each disease was: IBD n=225, Alzheimers n=99, MS n=71, T1D n=40. Enrichment fold changes were calculated as the log of the actual number of SNPs with significant disease signal (dependent on level) minus the log of the mean number of SNPs based on the permuted data. Enrichment P-values were calculated by counting the number of permutations with greater than or equal to the actual number of SNPs with significant disease signal plus one, divided by 1000 (number of permutations).

Patient Genotypes

The Sanger imputation service[13-16] was used to impute the patient single nucleotide positions (SNPs) from the results of the genotyping array using the UK10K+1000 Genomes Phase 3/Haplotype Reference Consortium (release 1.1) reference panel (Jan 2016). We discarded variants with an allele frequency of less than 5%, resulting in 5,323,884 SNPs. 202 of the 236 IBD risk SNPs[27,29] were imputed and PLINK[33] was used to calculate genetic risk scores. The genetic risk scores were used to predict disease for this patient in diagnostic model analyses performed as part of Figure 6 A and B.

Diagnostic Classification Models

Random forest classification models were built for each dataset (RNAseq / DNAm /16S data per gut segment) using diagnosis levels (disease vs. control, CD vs. UC) as the outcome measure using only the omics data and no covariates from the clinical data (Figure 6A). The maximum number of cross-validations (CVs) was determined by the sample size of each dataset (DNAm: all n=41, IBD only n=24; RNAseq: all n=32, IBD only =20; 16S: all n=63; IBD only n=42). Only autosomes were included in the model input data. The variance of each omics dataset was calculated and data points with low variance (<10%) were removed from the datasets as a method of feature selection. The models were built using the number of samples and features present in each dataset, 1000 estimators, no maximum depth and a minimum split of two samples. Each model was trained on the data using shuffle split CV and feature selection, with 30% of samples used as the test set for each CV. Results from the cross validation were used to assess the area under the curve (AUC), accuracy, precision, sensitivity and specificity.

Additionally, for the DNAm model, we tested the model performance using the independent follow-up data (Figure S8B).

Weighted gene correlation network analysis (WGCNA)

WGCNA was used to investigate the association between the omics data layers and disease outcome based several important clinical variables (Table S1). The general process of WGCNA is the clustering of the data points into modules to reduce the dimensions of the dataset. The use of modules (groups of genes/CpGs) greatly relieves the necessity of multiple testing as the number of tests performed is not based on the size of the omics dataset, rather the number of clinical parameters and modules. Each data point within the module has a 'gene significance score' which is the –log(p-value). A mean module gene significance is then calculated and this is correlated to the clinical parameter using Pearson's correlation method. WGCNA was performed for each diagnosis (CD and UC) by gut segment and dataset e.g CD samples from the SC using gene expression data. For the few missing values in this clinical data the median value was used for the patient group to complete the dataset. M-values were used for DNAm and r-log normalised gene counts were used for gene expression data. The *WGCNA* workflow as outlined in WGCNA tutorials: (https://labs.genetics.ucla.edu/horvath/CoexpressionNetwork/Rpackages/WGCNA/Tutorials/) was implemented using the WGCNA R package[34,35] The top 10% of varying data points (CpG sites or gene reads) for each dataset were used in this analysis (DNAm n=37,421, RNAseq n=5,488). The most suitable power setting was chosen for each dataset based on the Scale Independence and Mean Connectivity plots (based on model fit >0.8 and point of plateau on the graph). Module size was chosen to result in approximately 30/40 modules. Correlation analysis was performed between each module each clinical variable (Pearson) and p-values were calculated. Modules with a correlation (based on the eigengene) >±0.6 and p-value<0.05 were analysed further. Intramodular scatter plots were created and modules were annotated for significantly correlated traits. Heatmaps were generated based on the top modules and used to categorise patients into two groups of IBD patients. These groups were then used to plot Kaplan-Meier curves (*survival* R module; survFit function) based on the top correlating clinical variables. Finally, the annotation of the top modules was compared between the DNAm and RNAseq data to look at the similarity between the data layers and clinical variable outcome association.

References

1. Levine A, Koletzko S, Turner D, et al. ESPGHAN revised porto criteria for the diagnosis of inflammatory bowel disease in children and adolescents. J Pediatr Gastroenterol Nutr 2014;58:795–806.

2. Van Assche G, Dignass A, Panes J, et al. The second European evidence-based Consensus on the diagnosis and management of Crohn's disease: Definitions and diagnosis. J Crohns Colitis 2010;4:7–27.

3. Stange EF, Travis SPL, Vermeire S, et al. European evidence-based Consensus on the diagnosis and management of ulcerative colitis: Definitions and diagnosis. J Crohns Colitis 2008;2:1–23.

4. Jenke AC, Postberg J, Raine T, et al. DNA methylation analysis in the intestinal epithelium-effect of cell separation on gene expression and methylation profile. PLoS One 2013;8:e55636.

5. Kraiczy J, Nayak K, Ross A, et al. Assessing DNA methylation in the developing human intestinal epithelium: potential link to inflammatory bowel disease. Mucosal Immunol 2016;9:647–658.

6. Häsler R, Sheibani-Tezerji R, Sinha A, et al. Uncoupling of mucosal gene regulation, mRNA splicing and adherent microbiota signatures in inflammatory bowel disease. Gut 2016. Available at: http://dx.doi.org/10.1136/gutjnl-2016-311651.

7. Aryee MJ, Jaffe AE, Corrada-Bravo H, et al. Minfi: a flexible and comprehensive Bioconductor package for the analysis of Infinium DNA methylation microarrays. Bioinformatics 2014;30:1363–1369.

8. Leek JT, Johnson WE, Parker HS, et al. The sva package for removing batch effects and other unwanted variation in high-throughput experiments. Bioinformatics 2012;28:882–883.

9. Peters TJ, Buckley MJ, Statham AL, et al. De novo identification of differentially methylated regions in the human genome. Epigenetics Chromatin 2015;8:6.

10. Ritchie ME, Phipson B, Wu D, et al. limma powers differential expression analyses for RNA-sequencing and microarray studies. Nucleic Acids Res 2015;43:e47.

11. Hahne F, Ivanek R. Visualizing Genomic Data Using Gviz and Bioconductor. In: Mathé E, Davis S, eds. *Statistical Genomics: Methods and Protocols*. New York, NY: Springer New York; 2016:335–351.

12. Hansen K D AM. *IlluminaHumanMethylation450kmanifest: Annotation for Illumina's 450k methylation arrays. R package version 0.4.0*. 2012.

13. Martin M. Cutadapt removes adapter sequences from high-throughput sequencing reads. EMBnet.journal 2011;17:10–12.

14. Kim D, Pertea G, Trapnell C, et al. TopHat2: accurate alignment of transcriptomes in the presence of insertions, deletions and gene fusions. Genome Biol 2013;14:R36.

15. Langmead B, Salzberg SL. Fast gapped-read alignment with Bowtie 2. Nat Methods 2012;9:357–359.

16. Li H, Handsaker B, Wysoker A, et al. The Sequence Alignment/Map format and SAMtools. Bioinformatics 2009;25:2078–2079.

17. Anders S, Pyl PT, Huber W. HTSeq--a Python framework to work with high-throughput sequencing data. Bioinformatics 2015;31:166–169.

18. Love MI, Huber W, Anders S. Moderated estimation of fold change and dispersion for RNA-seq data with DESeq2. Genome Biol 2014;15:550.

19. Vázquez-Baeza Y, Pirrung M, Gonzalez A, et al. EMPeror: a tool for visualizing high-throughput microbial community data. Gigascience 2013;2:16.

20. DeSantis TZ, Hugenholtz P, Larsen N, et al. Greengenes, a chimera-checked 16S rRNA gene database and workbench compatible with ARB. Appl Environ Microbiol 2006;72:5069–5072.

21. McDonald D, Price MN, Goodrich J, et al. An improved Greengenes taxonomy with explicit ranks for ecological and evolutionary analyses of bacteria and archaea. ISME J 2012;6:610–618.

22. Price MN, Dehal PS, Arkin AP. FastTree 2–approximately maximum-likelihood trees for large alignments. PLoS One 2010. Available at: http://journals.plos.org/plosone/article?id=10.1371/journal.pone.0009490.

23. Edgar RC. Search and clustering orders of magnitude faster than BLAST. Bioinformatics 2010;26:2460–2461.

24. McMurdie PJ, Holmes S. phyloseq: an R package for reproducible interactive analysis and graphics of microbiome census data. PLoS One 2013;8:e61217.

25. Breuer K, Foroushani AK, Laird MR, et al. InnateDB: systems biology of innate immunity and beyond--recent updates and continuing curation. Nucleic Acids Res 2013;41:D1228–33.

26. Sato T, Stange DE, Ferrante M, et al. Long-term expansion of epithelial organoids from human colon, adenoma, adenocarcinoma, and Barrett's epithelium. Gastroenterology 2011;141:1762–1772.

27. Jostins L, Ripke S, Weersma RK, et al. Host-microbe interactions have shaped the genetic architecture of inflammatory bowel disease. Nature 2012;491:119–124.

28. Huang H, Fang M, Jostins L, et al. Association mapping of inflammatory bowel disease loci to single variant resolution. bioRxiv 2015:028688. Available at: http://biorxiv.org/content/early/2015/10/20/028688 [Accessed July 27, 2017].

29. Lange KM de, Moutsianas L, Lee JC, et al. Genome-wide association study implicates immune activation of multiple integrin genes in inflammatory bowel disease. bioRxiv 2016:058255. Available at: http://www.biorxiv.org/content/early/2016/06/10/058255 [Accessed July 25, 2017].

30. Queralt-Rosinach N, Piñero J, Bravo À, et al. DisGeNET-RDF: harnessing the innovative power of the Semantic Web to explore the genetic basis of diseases. Bioinformatics 2016;32:2236–2238.

31. Piñero J, Queralt-Rosinach N, Bravo À, et al. DisGeNET: a discovery platform for the dynamical exploration of human diseases and their genes. Database 2015;2015:bav028.

32. Pers TH, Timshel P, Hirschhorn JN. SNPsnap: a Web-based tool for identification and annotation of matched SNPs. Bioinformatics 2015;31:418–420.

33. Purcell S, Neale B, Todd-Brown K, et al. PLINK: a tool set for whole-genome association and population-based linkage analyses. Am J Hum Genet 2007;81:559–575.

34. Langfelder P, Horvath S. WGCNA: an R package for weighted correlation network analysis. BMC Bioinformatics 2008;9:559.

35. Zhang B, Horvath S. A general framework for weighted gene co-expression network analysis. Stat Appl Genet Mol Biol 2005;4:Article17.

36. Sing T, Sander O, Beerenwinkel N, et al. ROCR: visualizing classifier performance in R. Bioinformatics 2005;21:3940–3941.
