# Supplementary material for: DNA Methylation and Transcription Patterns in Intestinal Epithelial Cells From Pediatric Patients With Inflammatory Bowel Diseases Differentiate Disease Subtypes and Associate With Outcome
Source: Gastroenterology. 2018 Feb;154(3):585–98. doi: 10.1053/j.gastro.2017.10.007 (PMC6381389; doi:10.1053/j.gastro.2017.10.007)
Supplement: Supplementary Figures S1–S9 [file mmc2.pdf]

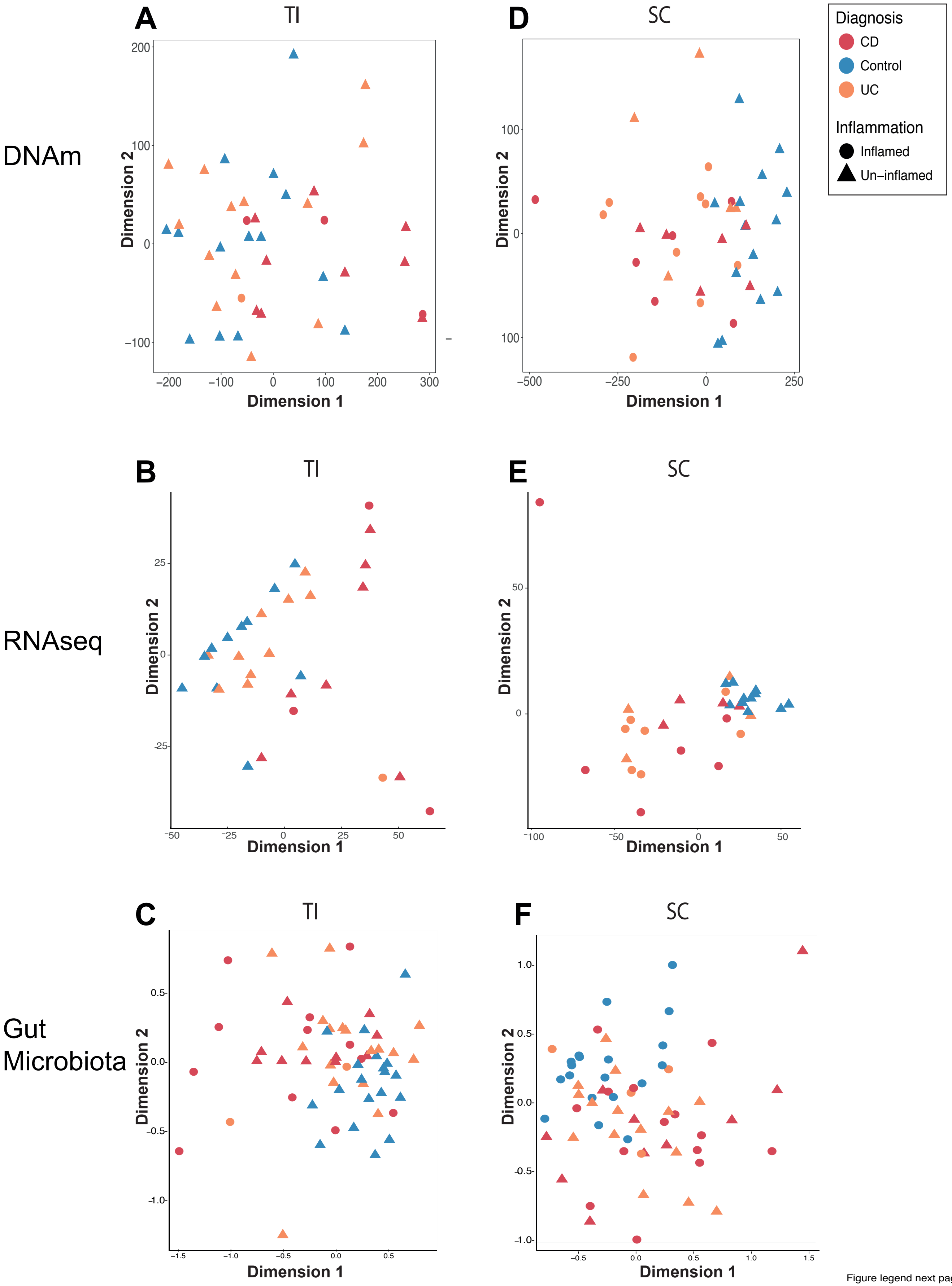

Figure legend next page

Figure S1: MDS plots for each dataset and gut segment - Ileum (TI) and Colon (SC) - labelled by diagnosis and inflammation (presence or absence). A) DNAm based on batch corrected M-values from the TI B) r-log normalised RNAseq gene expression counts from the TI C) gut microbiota 16S Operational Taxonomic Units (OTU) normalised counts from the SC. D) DNAm based on batch corrected M-values from the SC E) r-log normalised RNAseq gene expression counts from the SC F) gut microbiota 16S OTUs normalised counts from the SC.

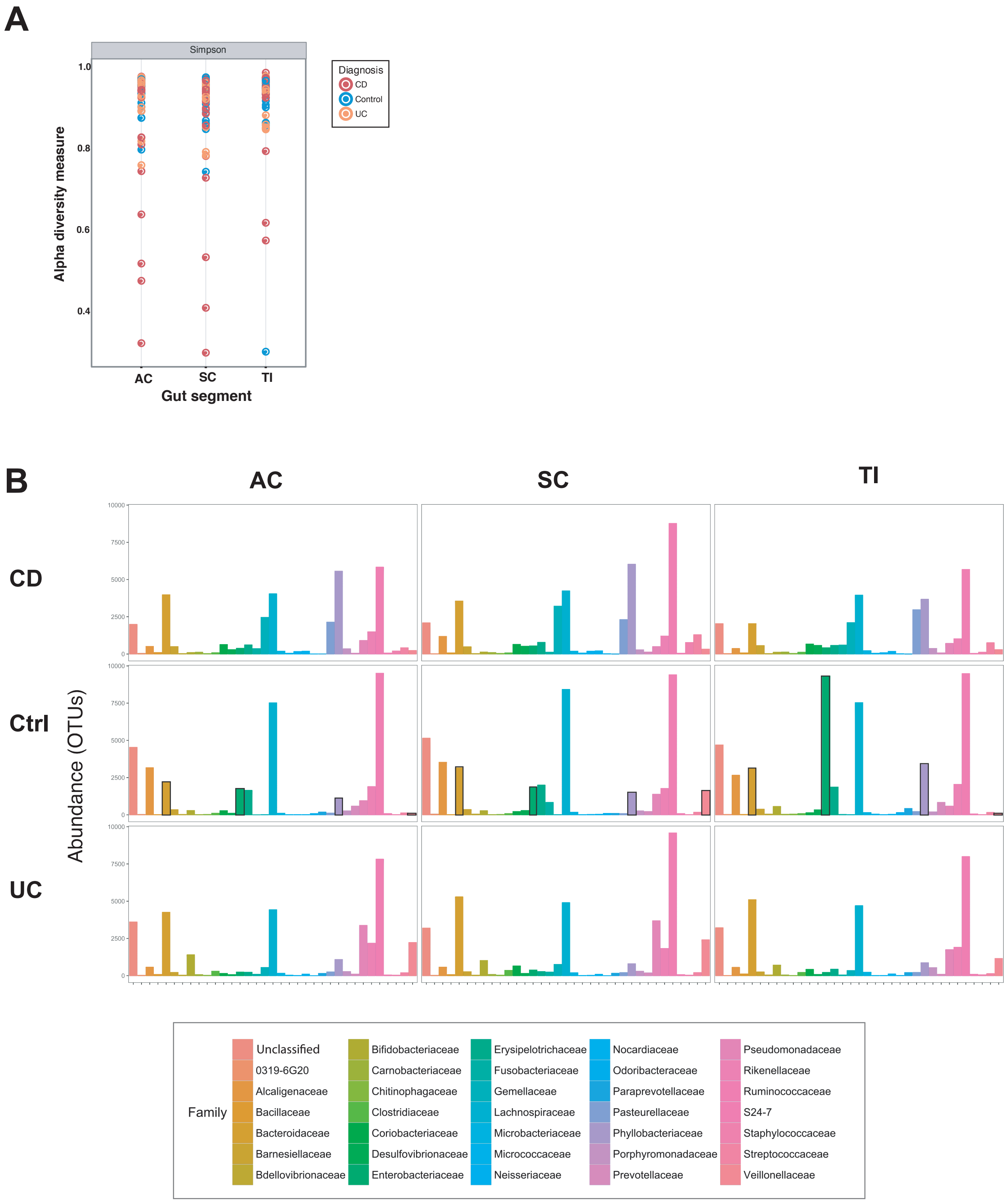

Figure S2: Summary of mucosa associated gut microbiota 16S operational taxonomic units (OTUs) analyses. A) Simpson's alpha diversity per sample by gut segment and diagnosis. Reduced diversity is present primarily in CD derived samples. B) Summary of the abundance of OTUs at the family level for each gut segment and diagnosis.

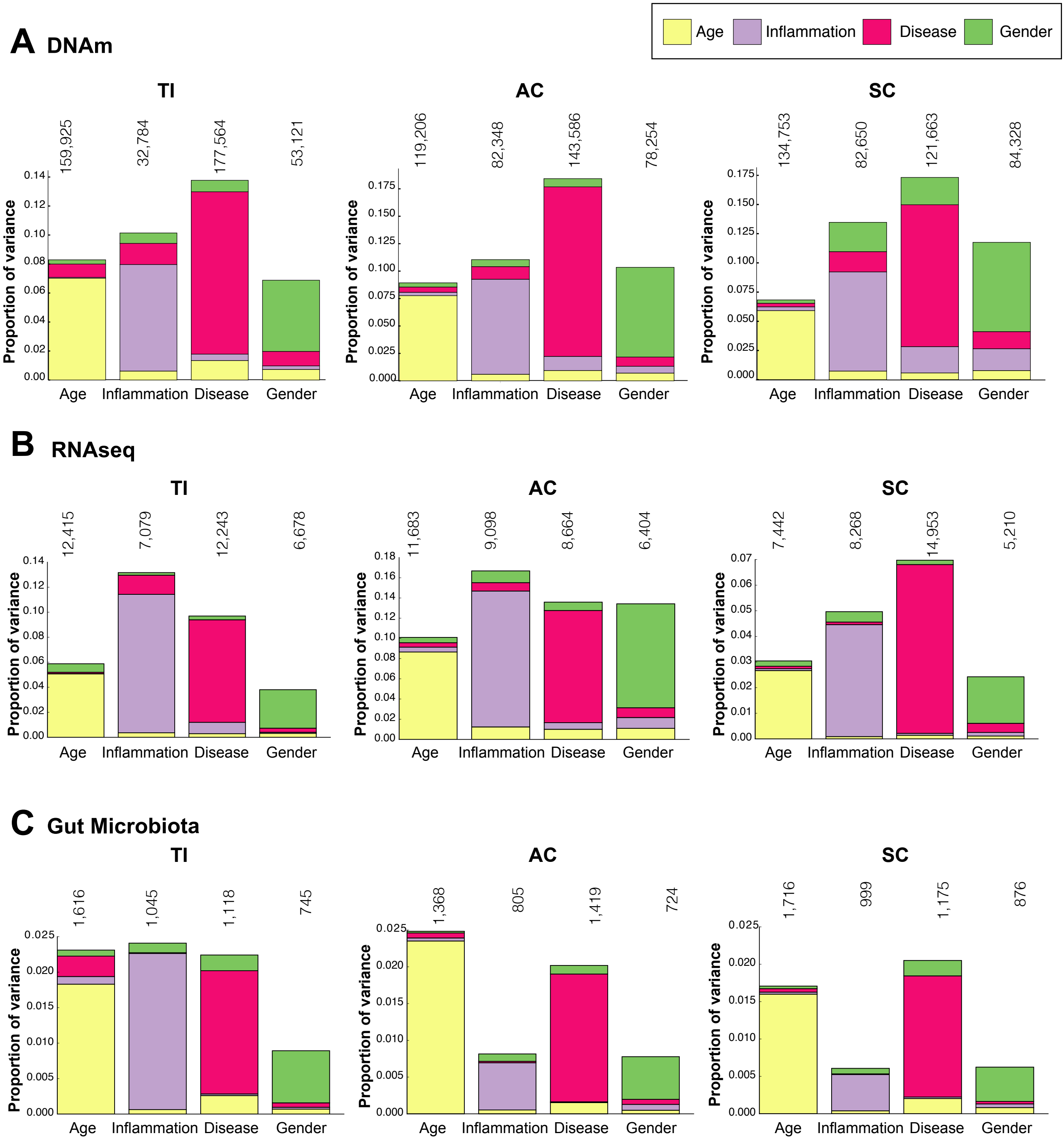

Figure S3: Bar charts of explained variance by gut segment and dataset. Each bar chart represents the analysis across the full dataset excluding the X and Y chromosomes. DNA methylation (DNAm) results were based on batch corrected M-values, RNAseq data based on the r-log normalised read counts and the gut microbiota based on normalised OTU counts. Each bar chart is labeled with the number of data points where the phenotype in question is the lead. Each bar is split between multiple phenotypes as multiple phenotypes can affect a single data point e.g. CpG site or gene.

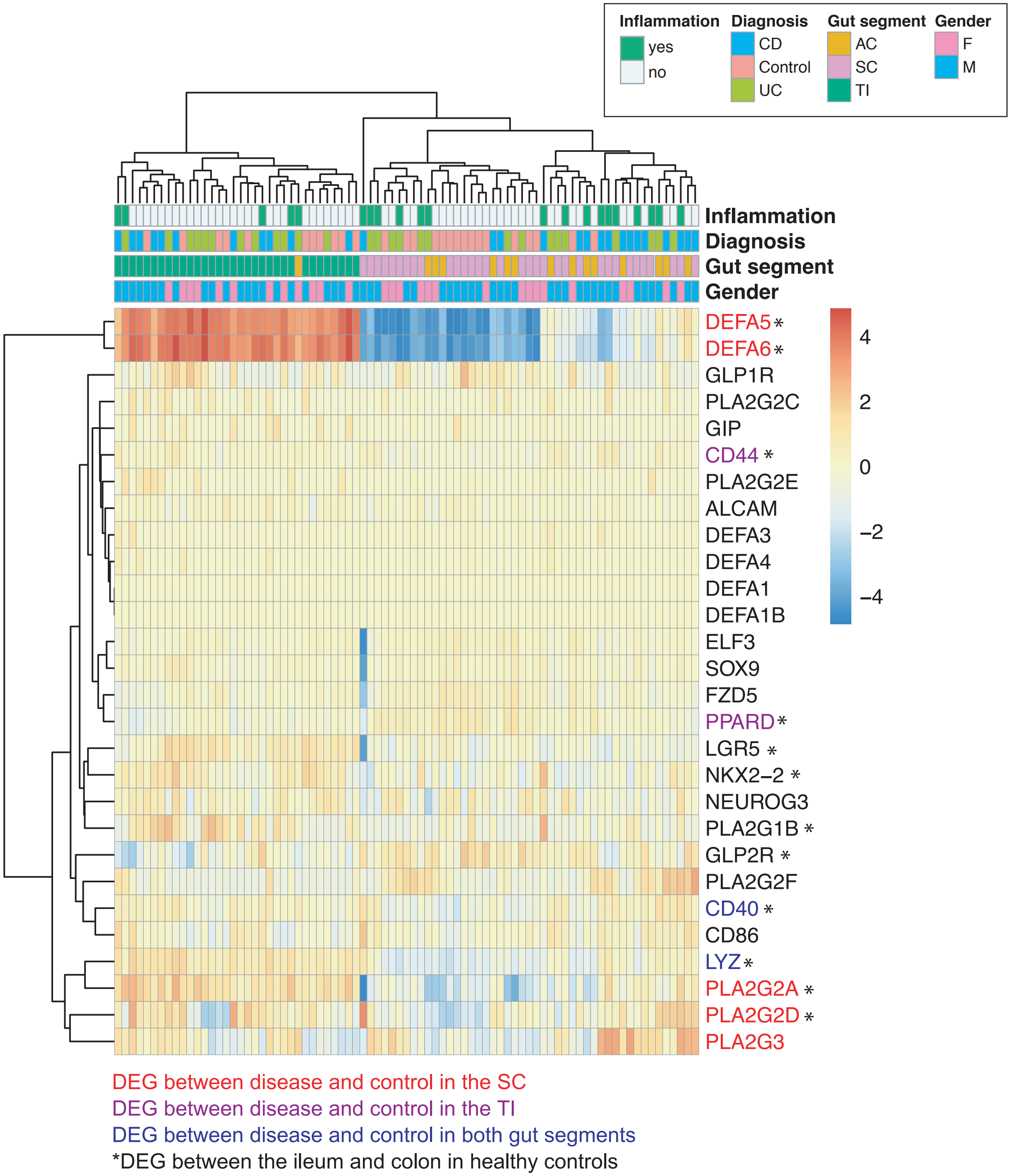

Figure S4: Heatmap of epithelial cell subtype and immune gene markers across gut segments and diagnoses. Heat map built using r-log normalized gene expression read count of common cell type markers. *Genes that are differentially expressed (DEGs) between the gut segments in healthy controls. DEGs between disease and control in the colon (SC) are labeled in red. DEGs between disease and control in the ileum (TI) are labeled in purple. DEGs between disease and control in both gut segments are labeled in blue.

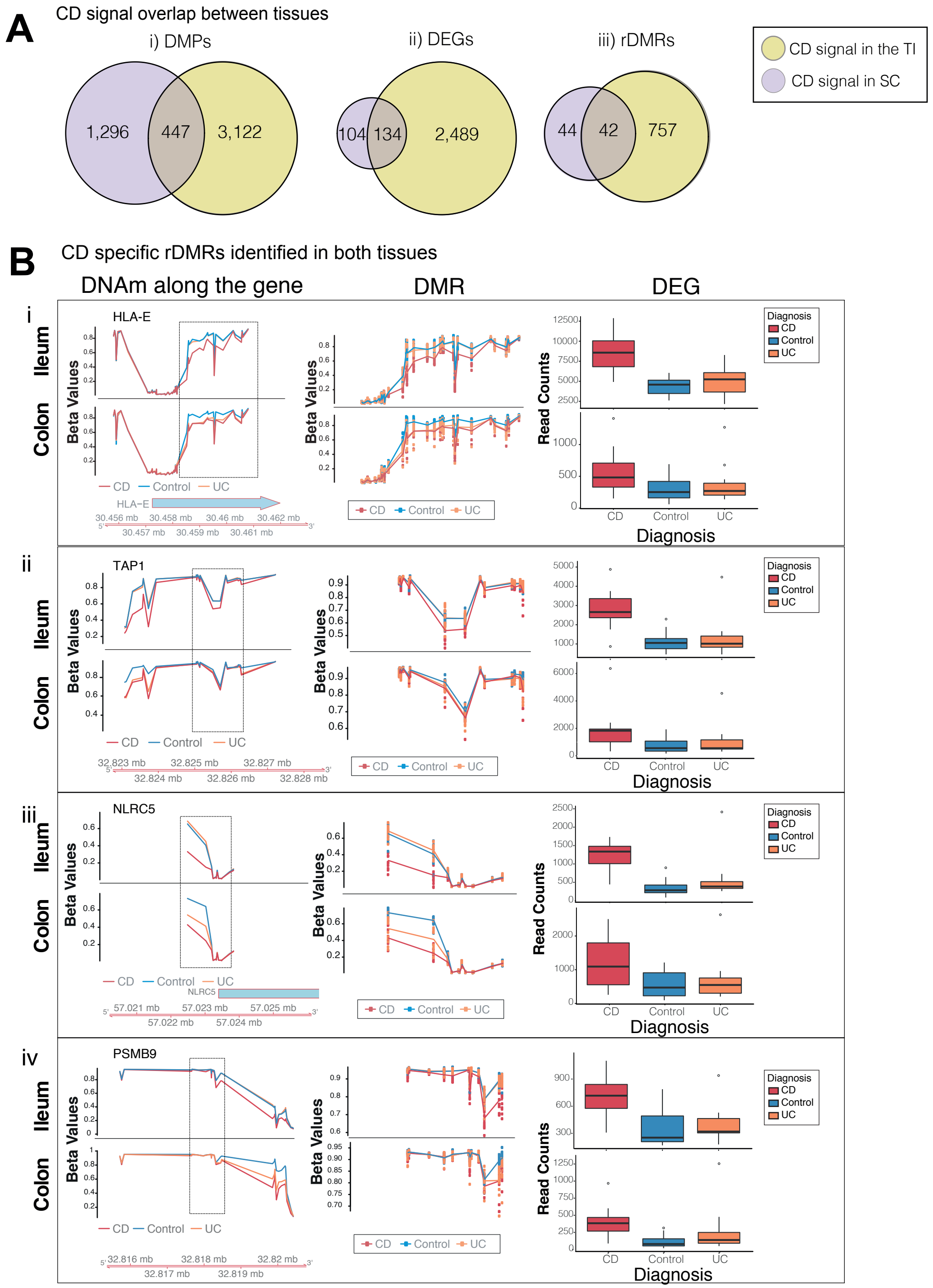

Figure S5: Crohn's disease DNAm and gene expression analysis overlap between gut segments. Differential DNAm and gene expression analysis were performed separately for TI (A and B) and SC (C and D), taking mucosal inflammation into account. Ai-iii) Venn diagrams of significant DMPs, DEGs and regulatory DMRs (rDMRs). Bi-iv) Examples of CD-specific rDMRs displaying DNA methylation levels expressed as Beta value on the y-axis in the left panel separately for TI and SC samples in the upper and lower panel respectively. Beta value of 0 represents un-methylated, while 1 represents fully methylated CpG site. Genomic coordinates are displayed on the x-axis. The middle panel displays identified rDMR (enlarged). The right panel displays a boxplot of the respective gene expression according to diagnosis i) HLA-E, ii) TAP1, iii) NLRC5 iv) PSMMB9.

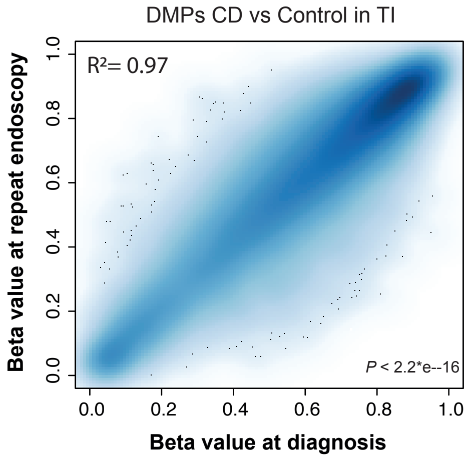

Figure S6: Correlation density plot of DNA methylation of the CD-associated epigenetic differences (DMPs) at time of diagnosis and time of repeat endoscopy. Plotted are beta values of TI epithelium in each CD patient that underwent repeat endoscopy. Intensity of the colour indicates higher density of data points. Correlation R2= 0.973, P < 2.2e-16 based on DMPs= 3569.

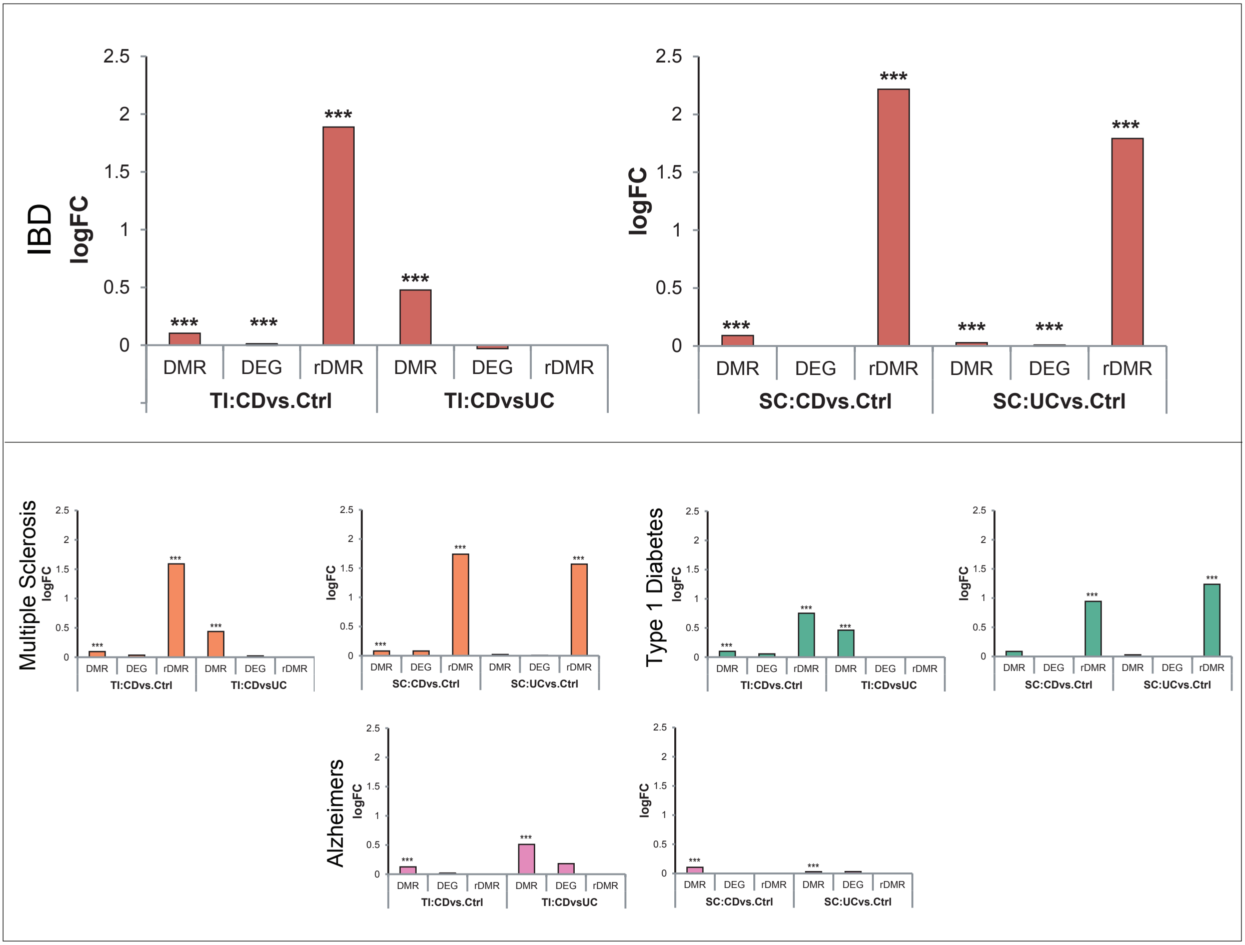

Figure S7: Enrichment analysis of disease-specific IEC DNAm and gene expression around genetic susceptibility loci. Enrichment analysis was performed for CD and UC specific DMRs, DEGs and rDMRs against a number of GWAS identified susceptibility loci predisposing to the development of, IBD (upper panel) as well as other diseases (Alzheimer's, Multiple Sclerosis (MS) and Type 1 Diabetes, lower panel). Results are displayed as log fold change (logFC) of number of SNPs with at least one DMR/DEG/rDMR within a set window, compared to the average from 1,000 permutations (i.e. random SNPs). Statistically significant enrichment is indicated according to p-value calculated based on 1,000 permutations: ***P < .001.

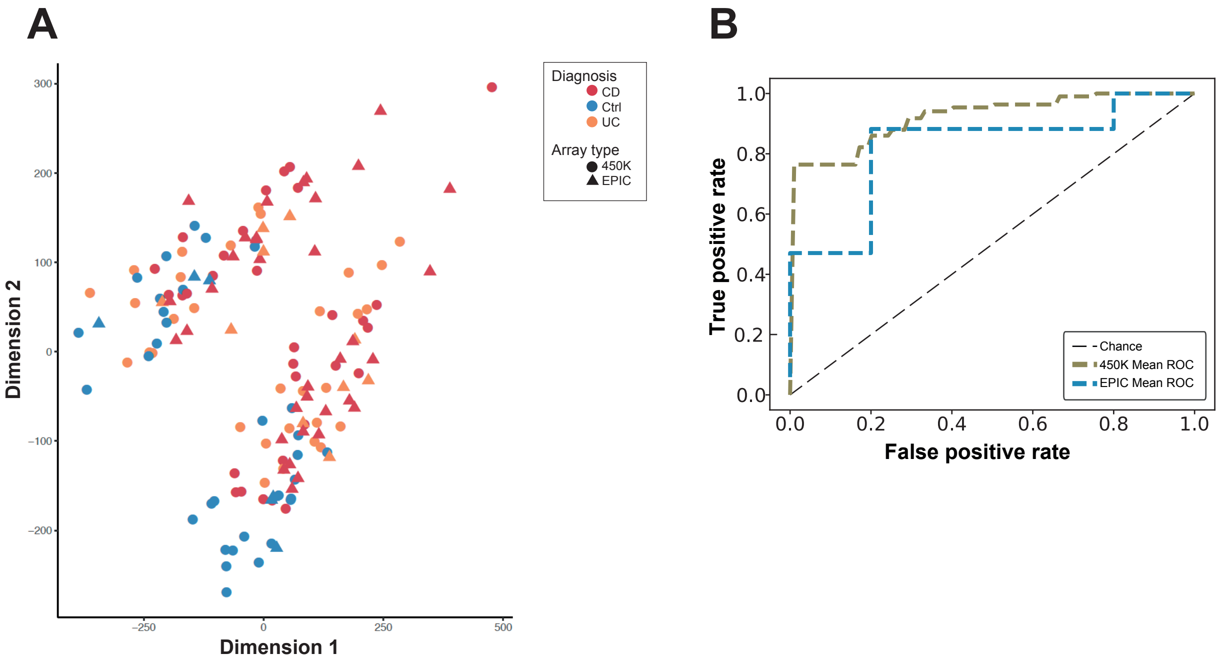

Figure S8: Extended sample set of intestinal epithelial DNA methylation profiles and validation of their diagnostic prediction in the random-forest model. An additional sample set of purified intestinal epithelium was obtained and genome-wide DNAm profiles generated using Illumina EPIC bead arrays. A) MDS plot of genome-wide DNAm profiles of samples measured by 450K (original/main sample set) and EPIC (additional samples) array. MDS plot is based on M-values of CpGs present in both arrays after batch-correction, autosomes only. B) ROC curve of the TI IBD model (to separate CD from UC) using DNA methylation data from the 450K array, validated in an additional cohort using samples profiled on EPIC array platform (AUC=0.82), see also Fig. 6Bii.

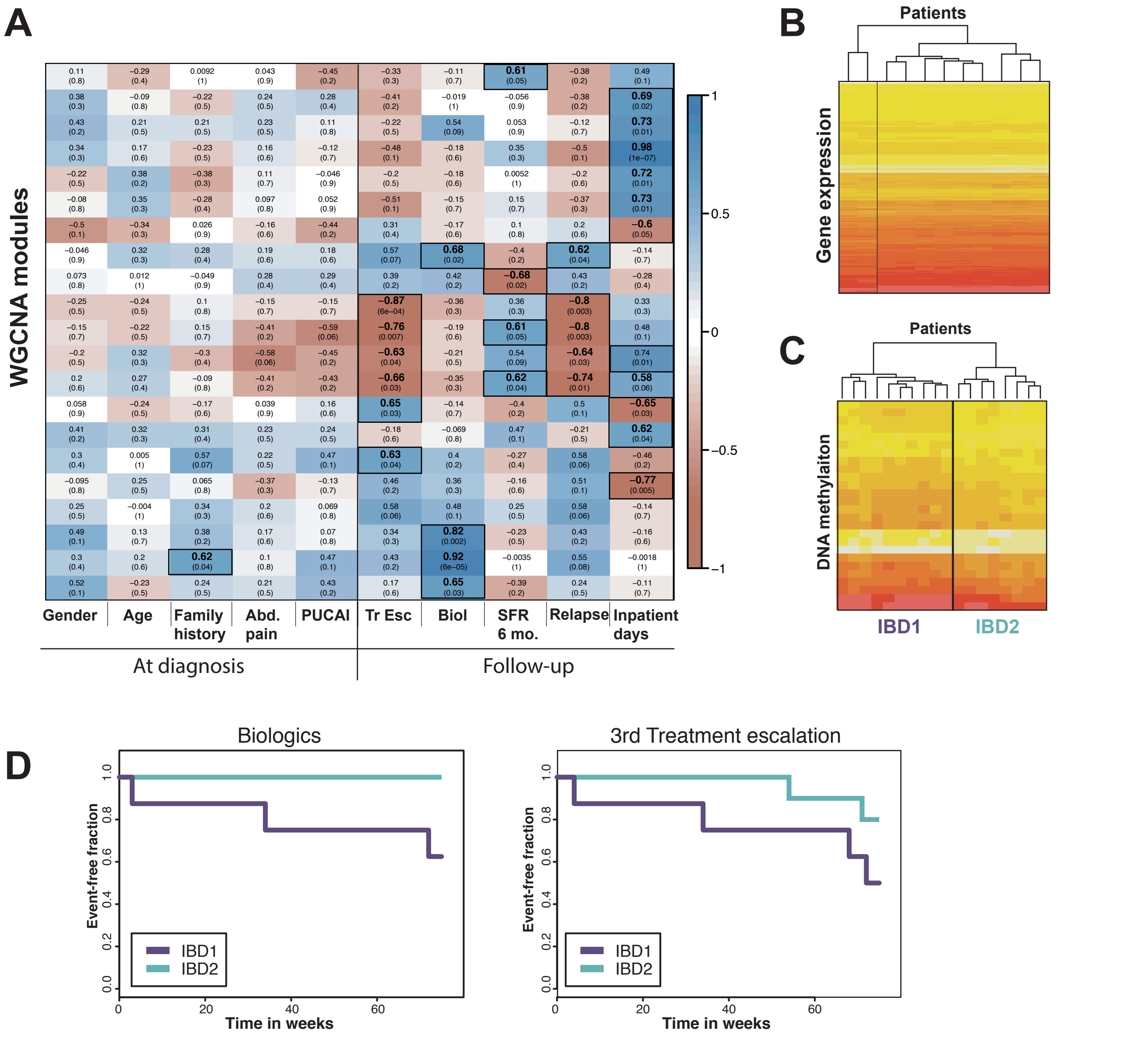

Figure S9: Weighted Gene Co-Expression Network Analysis (WGCNA) of SC gene expression and DNA methylation profiles from UC patients and correlation with clinical outcomes. A) Relationship heatmap of key gene expression modules (n=11 patients) and clinical parameters. Each cell displays the Pearson correlation coefficient and the corresponding p-value. B) Heat-map of the RNA-Seq data prognostic signature of top WGNA module for SC UC samples. Rows represent the gene expression pattern of each gene within the top module across the patient samples. The clustering of the heatmap separated the patients into two groups, which is indicated on the heatmap. C) Heat-map DNA methylation prognostic signature top WCGNA module of SC samples (n=18 patients). D) Kaplan- Meier curves for i) time to use of biologics and ii) time until third treatment escalation. Samples were separated according to the top module DNA methylation signature from C). Clinical data was collected during the 75-week follow-up (n=18 patients, log-rank test, *P*= .037 and *P*= .173). Age= Age at diagnosis, Abd.pain= Abdominal pain, PUCAI= Paediatric UC activity index, Tr Esc= Treatment escalations, Biol= Use of biologics, SFR= Steroid-free remission at 6 months, Inpatient days= Unplanned inpatient days.
